# An HBV‐Derived Peptide Poly6 as a Novel Candidate for Functional Cure Via IFN‐I–Mediated Epigenetic Regulation of cccDNA

**DOI:** 10.1002/jmv.70877

**Published:** 2026-03-27

**Authors:** Junghwa Jang, Dong Hyun Kim, Ziyun Kim, Eunseo Kim, Yu‐Min Choi, Bum‐Joon Kim

**Affiliations:** ^1^ Department of Microbiology and Immunology College of Medicine, Seoul National University Seoul Republic of Korea; ^2^ Department of Biopharmaceutical Engineering Hannam University Daejeon Republic of Korea; ^3^ Department of Biomedical Sciences College of Medicine, Seoul National University Seoul Republic of Korea; ^4^ Liver Research Institute, College of Medicine Seoul National University Seoul Korea; ^5^ Cancer Research Institute, College of Medicine Seoul National University Seoul Korea; ^6^ Institute of Endemic Diseases Seoul National University Medical Research Center (SNUMRC) Seoul Republic of Korea; ^7^ BK21 FOUR Biomedical Science Project Seoul National University College of Medicine Seoul Republic of Korea; ^8^ Wide River Institute of Immunology Seoul National University Hongcheon Republic of Korea

**Keywords:** cccDNA, epigenetic regulation of cccDNA, hepatitis B virus (HBV), IFI16, Sp1, Type 1 interferon (IFN‐I)

## Abstract

Covalently closed circular DNA (cccDNA) represents the central obstacle to achieving a functional cure for chronic hepatitis B virus (HBV) infection. Poly6, a peptide encoded within the HBV genome, was investigated for antiviral efficacy in hepatocyte‐derived cell lines, hydrodynamic injection models, and HBV transgenic mice. Poly6 administration markedly decreased cccDNA, pregenomic RNA, and viral DNA without detectable cytotoxicity. Poly6 also showed synergistic antiviral effects with entecavir. Mechanistic analyzes demonstrated that Poly6 initiates parallel upstream events: mitochondrial stress resulting in oxidized mtDNA release and activation of the STING–IRF3 pathway, and induction of IFI16, a nuclear DNA sensor implicated in interferon regulation. Both signals converged on robust type I interferon (IFN‐I) production. The IFN‐I response subsequently promoted expression of canonical ISGs, including iNOS, which generated nitric oxide to disrupt nucleocapsid assembly. Concurrently, IFI16, whose abundance was further increased by interferon signaling, amplified IFN‐I production and imposed epigenetic silencing of cccDNA through Sp1 sequestration and histone hypoacetylation. Chromatin immunoprecipitation confirmed reduced acetylation of H3K27, H4K5, and H4K12 on cccDNA minichromosomes. These results delineate a unified IFN‐I–centered cascade in which Poly6 coordinates complementary antiviral activities, supporting its translational potential as a therapeutic candidate for durable HBV control.

## 1 Introduction

Despite the existence of a capable vaccine, HBV infection persists as a significant driver of unfavorable consequences of liver diseases, including hepatocellular carcinoma (HCC) and cirrhosis. The annual death toll caused by HBV‐related disease is estimated at 887,000 [1], and 15%‐25% of chronic HBV patients die from cirrhosis or HCC [2, 3, 4].

Nuclear‐located cccDNA is a serious hurdle for a complete cure and a key factor for HBV persistence [5], which serves as the transcriptional template and stable molecular reservoir for HBV RNAs [6]. Despite effective inhibition of viral replication, NAs cannot eliminate cccDNA, and long‐term treatment with NAs can cause drug‐resistant viral strains [7, 8]. Interferon‐α (IFN‐α) has been reported to induce noncytolytic clearance of cccDNA in HBV‐infected hepatocytes [9, 10], although its therapeutic use is constrained by significant adverse effects [11, 12, 13]. Challenges in current therapies have prompted a focus on cccDNA‐directed tactics, involving both epigenetic repression and host‐driven innovations [14, 15, 16, 17]. However, none have yet achieved clinical validation, leaving a significant therapeutic gap [18].

Interferon‐inducible protein 16 (IFI16) belongs to the absent in melanoma (AIM) 2‐like receptors family, that functions as a nuclear DNA sensor. It recognizes foreign or aberrant DNA within the nucleus and subsequently activates innate immune pathways, including the STING–IRF3 axis, to induce type I interferon (IFN‐I) responses [19]. In addition to its sensing activity, IFI16 expression itself can be further enhanced by interferon signaling, positioning it as both a regulator and a target within the IFN network. Importantly, IFI16 has been implicated in the transcriptional control of viral episomes through direct interaction with viral DNA and associated transcription factors, thereby contributing to epigenetic repression. These features suggest that IFI16 could play a key role in controlling hepatitis B virus (HBV) replication, particularly by influencing cccDNA activity.

HBV‐originated 6‐mer peptide, Poly6, was first reported to show antiviral activity against HIV‐1 infection by interfering viral Integrase activity, which is mediated by epigenetic modification [20]. It has also been reported to have a potent antiviral effect against SARS‐CoV‐2 infections in an IFN‐I ‐dependent manner [21]. In addition, we recently reported that vaccination of HBV surface antigen (HBsAg) combined with Poly6 exerted humoral and cell‐mediated immunity through dendritic cell (DC) activation in an IFN‐I‐dependent manner [22]. Collectively, these findings suggest the potential of Poly6 as an antiviral drug candidate against HBV capable of inhibiting viral cccDNA via IFN‐I signaling‐mediated epigenetic modification.

In this work, we sought to bridge the existing therapeutic gap by (i) assessing the antiviral potential of Poly6 using both in vitro and in vivo HBV models, and (ii) investigating its mechanism of action, particularly focusing on mtDNA stress–driven IFN‐I activation and the epigenetic regulation of cccDNA via IFI16/Sp1 interactions.

## 2 Materials and Methods

### 2.1 Selection of Peptides Derived From the HBV Polymerase Region

**Figure 1:**
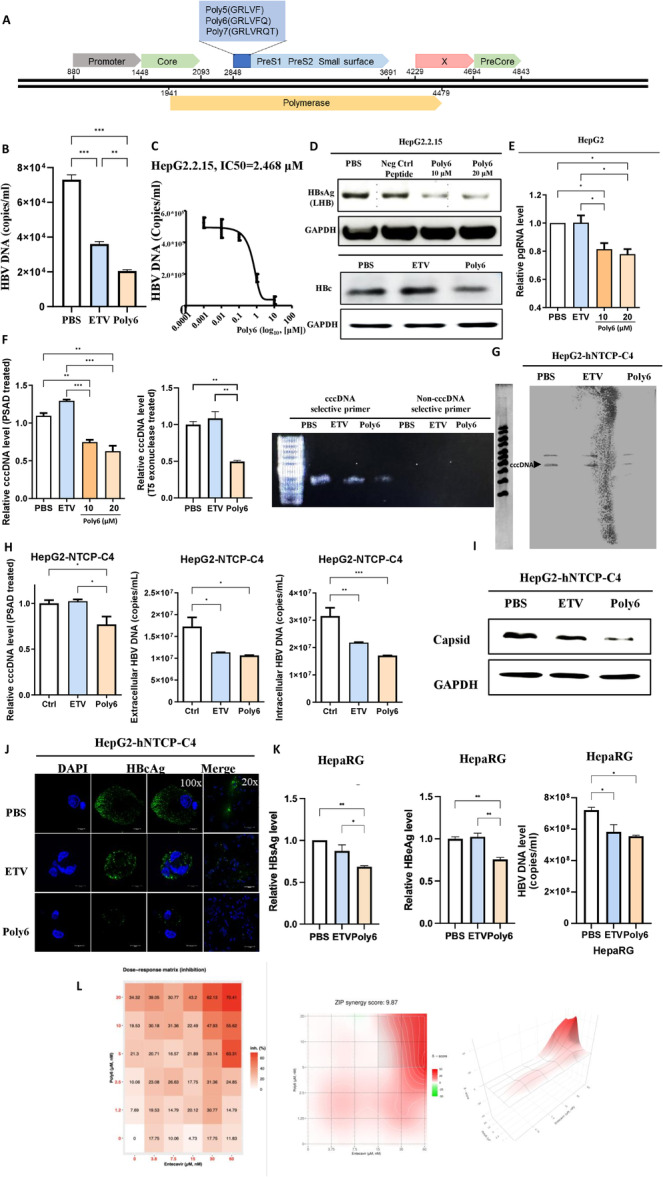
The originality of Poly6 and its anti‐HBV effects in human hepatoma cells. (A) Schematic representation of three peptides derived from the HBV polymerase gene region overlapping the ORF of the PreS1 region. (B and C) Extracellular HBV DNA and IC₅₀ of Poly6 in HepG2.2.15 cells after 48 h treatment (ETV; 30 nM, Poly6; 10 μM) (D) HBc and HBV large surface protein (LHB) detected by western blot, with a random peptide (FLGVRQ) as negative control. (E and F) 3.5‐kb pgRNA and cccDNA levels in HepG2 cells transfected with linearized 1.2× HBV genome. (G) Southern blot analysis of HepG2‐hNTCP‐C4 cells. (H) cccDNA levels, intra‐ and extracellular HBV DNA levels in HepG2‐hNTCP‐C4 cells (ETV; 30 nM, Poly6; 10 μM). (I) HBV core protein and capsid formation analyzed by western blot using SDS–PAGE and nonreducing native gels. (J) Immunofluorescence of HBcAg in HepG2‐hNTCP‐C4 cells. (K) HBsAg, HbeAg, and HBV viral DNA levels were measured in HepaRG primary cells (ETV; 30 nM, Poly6; 10 μM). (L) Synergistic antiviral effects were measured in HepG2.2.15 cells treated with ETV (up to 60 nM) and Poly6 (up to 20 μM) in the combination. The synergy score was analyzed using the Synergy Finder web application (version 3.0). Data indicate the mean ± S.D. of two to three independent experiments with 95% CI. *p < 0.05, **p < 0.01, ***p < 0.001.

We have previously reported that 5' preS1 region nucleotide (nt) deletions were mainly identified from occult HBV infection and hepatocellular carcinoma patients in South Korea, and those deletions were related to severe liver disease progression [23]. Because the large surface region overlaps with a polymerase gene in the HBV full genome, we developed three peptide designs from a polymerase corresponding to the 5' preS1 region deletions and denominated these peptides Poly5, Poly6, or Poly7 (Figure 1A).

To evaluate the antiviral effect of the three peptides, virion replication and HBsAg were estimated by quantitative PCR and ELISA after 48 h of treatment with phosphate‐buffered saline (PBS), entecavir (ETV, 30 nM) or one of the three peptides (10 μM) in HepG2.2.15 cells and HepG2 cells. Poly6 showed the best overall responses (Supporting Information Figure S1); therefore, the Poly6 was selected to investigate an anti‐HBV effect in this study.

### 2.2 Cells and Plasmids

The HepG2 cells (Korean Cell Line Bank, Seoul, Korea), which are human hepatocellular carcinoma cells, HepG2.2.15 cells (Sigma‐Aldrich, MO, USA) permanently integrated with the HBV construct, HepG2‐hNTCP‐C4 cells (kindly gifted by Dr. Koichi Watashi from National Institute of Infectious Disease (Tokyo, Japan) manifesting a NTCP receptor on the cell membrane, and hMH55‐293‐ISRE cells containing integrated interferon‐sensitive response element (ISRE) genes tagging luciferase reporter genes in chromosomes were used in this study. The pHBV‐1.2X‐Wildtype (Genotype C) or pCIDA‐HBV‐BCore‐HBc (Genotype D, kindly provided by Dr. Hu J et al.) plasmid was used for transfection for inducing acute infection using Lipofectamine 3000 reagent (Life Technologies).

### 2.3 Peptides and Reagents

GRLVFQ (Poly6, IC_50_ = 2.468 µM) peptide was synthesized by Peptron Inc. (Daejeon, Korea). Entecavir (ETV, SML1103, IC_50_ = 0.2–0.5 nM) [24] and Rotenone were purchased from Sigma‐Aldrich (St. Louis, MO, USA). Poly6 and ETV were dissolved in phosphate‐buffered saline (PBS) and used for all *in vitro* and *in vivo* experiments.

### 2.4 HBV cccDNA Chip Assay

ChIP assays were conducted using a ChIP Plus Enzymatic Chromatin IP kit (9003, CST, USA) according to the manufacturer's instruction. Briefly, cells were lysed after fixation, and chromatin was fragmented by Microcroccal Nuclease and sonication. Immunoprecipitation of chromatin is carried out using ChIP‐exclusive antibodies and Protein G magnetic beads. DNA purification is performed with DNA purification spin columns. Normalization is calculated as a percentage of input DNA using the formula ∆Ct = Ct (input)−Ct (immunoprecipitation).

### 2.5 Southern Blot Assay

HBV cccDNA was detected by Southern blotting of amplified products using the DIG DNA Labeling and Detection Kit (Roche, cat. #11093657910). cccDNA was extracted from HepG2.2.15 and HepG2‐NTCP‐C4 cells using the Hirt extraction method [25]. The extracted DNA was subjected to multiple displacement amplification with phi29 DNA polymerase (Thermo Fisher, cat. #EP0091) in the presence of Exo‐resistant random primers (Thermo Fisher, cat. #SO181) and dNTPs at 30°C for 30 min, followed by enzyme inactivation at 65°C for 4 h. The amplified DNA was then used as a template for PCR with the P1–P2 primer pair spanning the full‐length HBV genome, as described by Lebossé et al. [26]. Primer sequences are provided in Supporting Information Table S1. PCR products were resolved on a 1.2% agarose gel at 50 V and transferred to a nylon membrane (Hybond N + , Amersham, UK). The membrane was hybridized overnight at 42°C with a DIG‐labeled 1.2× wild‐type HBV full‐genome probe generated by XhoI digestion, followed by washing, incubation with an anti‐DIG antibody, and colorimetric detection using the reagents supplied with the kit.

### 2.6 Detection of HBV pgRNA

Total RNA was treated with RQ1 DNase (Promega, UK). For viral pgRNA detection, RNA was reverse transcribed to cDNA and detected by RT‐qPCR. To evaluate the 3.5 kbp of pgRNA, specific primers and probe were used. Human GAPDH and 18S rRNA genes were used for normalization.

### 2.7 HBV Infection

HBV virion‐containing supernatant was filtered and treated with 6% PEG‐8000. The supernatant was ultracentrifuged, and the virion was collected in PBS containing 15% FCS. For inoculation, HepG2‐hNTCP‐C4 cells were infected with the virion in the presence of 3% DMSO and 4% PEG8000 for 20 h.

### 2.8 Hydrodynamic Injection

10 μg of genotype C genome containing 1.2x‐WT plasmids were injected in the tail vein in male C57BL/6 mice (6‐8‐week) in a volume of saline of 10% of the mouse body weight. The plasmid‐containing saline was transferred within 5–8 s. All experimental methods were carried out in accordance with guidelines and regulations, and the animal study was approved by the Seoul National University Institutional Animal Care and Use Committee (IACUC) [SNU‐230622‐1‐1].

### 2.9 Transgenic Mouse (TG) Model

HBV TG mice were produced by Macrogen, Inc (Seoul, Korea) as previously described [22] [IACUC number SNU‐200918‐5‐2]. Male transgenic mice were injected with PBS, 0.1 mg/kg Entecavir (ETV), or 50 µg/kg Poly6 via intraperitoneal injection daily. At 4 and 8 weeks, the serum HBsAg and HBV DNA levels were analyzed by ELISA and quantitative PCR, respectively. The animal study was examined and confirmed by the Seoul National University Institutional Animal Care and Use Committee (IACUC) [SNU‐180530‐1‐2, SNU‐230103‐5‐2].

### 2.10 Statistical Analysis

Statistics were performed with the one‐way ANOVA method. The *p* value of significance was designated at *p* < 0.05 (*), 0.01 (**), or 0.001 (***). Data represent the mean ± S.D. of three independently performed experiments.

A detailed description of the materials and methods is provided in the Supporting Information S1.

## 3 Results

### 3.1 Cell Viability and Anti‐Viral Activity of Poly6 against HBV on In Vitro Systems

Prior to evaluating the anti‐HBV effect of Poly6, we tested for cytotoxicity to confirm that the anti‐HBV effect was independent of nonspecific cell toxicity. Results revealed that Poly6 did not induce significant cell cytotoxicity in HepG2, HepG2.2.15, and HepG2‐hNTCP‐C4 cells and did not affect cell viability, at concentrations up to 10 μM (Fig. S2).

Then, to verify the antiviral effect of Poly6, we applied Poly6 to the HepG2.2.15 permanent cells, which constantly express HBV virions. After treatment with PBS, ETV, or Poly6 for 24 h, the extracellular DNA level was remarkably decreased by Poly6 compared to PBS or ETV (Figure 1B). The mean IC_50_ of HBV by Poly6 was approximately 2.468 μM in the cells according to a dose‐dependent assay (Figure 1C). Furthermore, HBc and HBsAg protein expression decreased significantly in the Poly6‐treated group relative to both PBS and a scrambled peptide control (FLGVRQ) (Figure 1D and Supporting Information S3). As the HepG2.2.15 cells are derived from HepG2 cells and release HBV genotype D virion [27], we also examined the anti‐viral effect of Poly6 in HepG2 cells, transiently transfected with pCIDA‐HBV‐BCore‐HBc (Genotype D) plasmid. Poly6 treatment markedly reduced HBsAg, HBV DNA, and HBcAg compared with controls (Supporting Information Figure S4). To verify the specificity of its antiviral effect, we examined whether Poly6 affects the expression of non‐HBV proteins. Poly6 treatment did not alter GFP expression following transient transfection (Supporting Information Figure S5), indicating that its inhibitory activity is specific to HBV replication rather than a general suppression of plasmid‐derived protein expression. Further, in cells transfected with the 1.2× WT HBV genome (genotype C), Poly6 also lowered pgRNA levels (Figure 1E). Notably, Poly6 treatment reduced cccDNA levels in linearized 1.2× HBV‐transfected HepG2 cells, and this decrease was consistently observed after PSAD digestion and also under more stringent DpnI/T5 exonuclease treatment, which was further validated by electrophoresis assays (Figure 1F).

These results indicate that the antiviral mechanism induced by Poly6 under intracellular conditions may differ from that induced by ETV, which is a potent HBV polymerase inhibitor that becomes an active triphosphate form [28].

### 3.2 Antiviral Effects of Poly6 Against HBV in the HBV Infection System and Synergistic Effects

To assess the antiviral effect of Poly6 in an HBV infection model, HepG2‐hNTCP‐C4 cells expressing the sodium taurocholate cotransporting polypeptide (NTCP) receptor [29] were infected with HBV virions. Genotype‐C‐HBV‐infected HepG2‐hNTCP‐C4 cells were treated with PBS, ETV, or Poly6 for 72 h. Poly6 markedly reduced cccDNA levels compared with PBS or ETV, as confirmed by Southern blot and RT‐qPCR (Figure 1G,H). Consistently, both intra‐ and extracellular HBV DNA levels were remarkably reduced in ETV‐ and Poly6‐treated cells compared with PBS (Figure 1H right panel). Poly6 also suppresses nucleocapsid assembly and HBV core antigen expression, as shown by Western blot and immunofluorescence (Figure 1I–J).

Furthermore, in HBV‐infected primary HepaRG cells, Poly6 treatment reduced HBsAg, HBeAg, and HBV viral DNA secretion compared with PBS or ETV treatment (Figure 1K). These results further support the antiviral activity of Poly6 against HBV in a physiologically relevant, differentiated hepatocyte system. Co‐treatment of Poly6 with ETV in HepG2.2.15 cells resulted in a further decrease in HBsAg secretion and demonstrated synergistic inhibition of HBV replication as shown by a positive ZIP synergy score (9.87) (Figure 1L).

### 3.3 Poly6 Exhibits In Vivo Antiviral Activity Against HBV

**Figure 2:**
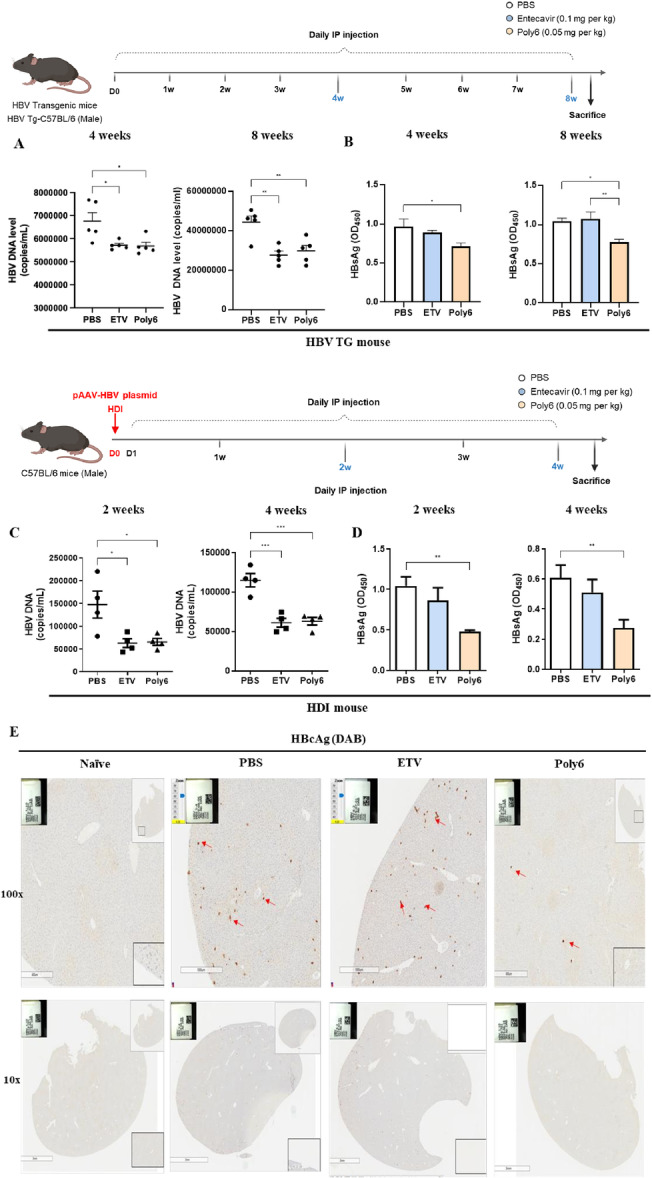
Antiviral activity of Poly6 against HBV in vivo. (A–B) Male HBV transgenic mice (*n* = 5 per group) were treated with PBS, entecavir (ETV, 0.1 mg/kg), or Poly6 (50 µg/kg), and serum HBV DNA (A) and HBsAg (B) levels were measured. (C–D) C57BL/6 mice were hydrodynamically injected with pAAV/HBV1.2 plasmids and treated intraperitoneally with PBS, entecavir (ETV, 0.1 mg/kg), or Poly6 (50 µg/kg). Serum HBV DNA (C) and HBsAg (D) levels were analyzed. (E) HBcAg expression in liver tissues detected by immunohistochemistry (100× magnification). Data are mean ± SD of three independent experiments with 95% CI. *p < 0.05, **p < 0.01, ***p < 0.001.

To evaluate the anti‐viral activity of Poly6 on the *in vivo* system, we injected PBS, entecavir (ETV, 0.1 mg/kg), or Poly6 (0.05 mg/kg) into HBV TG mice. HBV DNA levels were markedly reduced in the Poly6‐ or ETV‐treated groups compared with PBS controls at both 4 and 8 weeks (Figure 2A–B). Of note, a significant reduction in HBsAg was evident only in the Poly6‐treated mice. To further confirm the antiviral efficacy of Poly6 in an independent infection model, we employed hydrodynamic injection of pAAV/HBV1.2 plasmids into C57BL/6 mice [30]. Consistent with the TG model, HBV DNA levels were reduced in both the ETV and Poly6 groups, whereas a pronounced decline in HBsAg was observed only in the Poly6‐injected mice at both 2 and 4 weeks (Figure 2C–D). Liver immunohistochemistry also showed that treatment with Poly6, but not with PBS or ETV, led to a marked reduction in the number of HBcAg‐positive hepatocytes (Figure 2E).

Our findings indicate that Poly6 shows antiviral activity by decreasing HBsAg, HBcAg, and HBV DNA levels in the *in vivo* TG mouse model and hydrodynamic injection mouse model.

### 3.4 Antiviral Effects of Poly6 Against Drug‐Resistant HBV Variants

**Figure 3:**
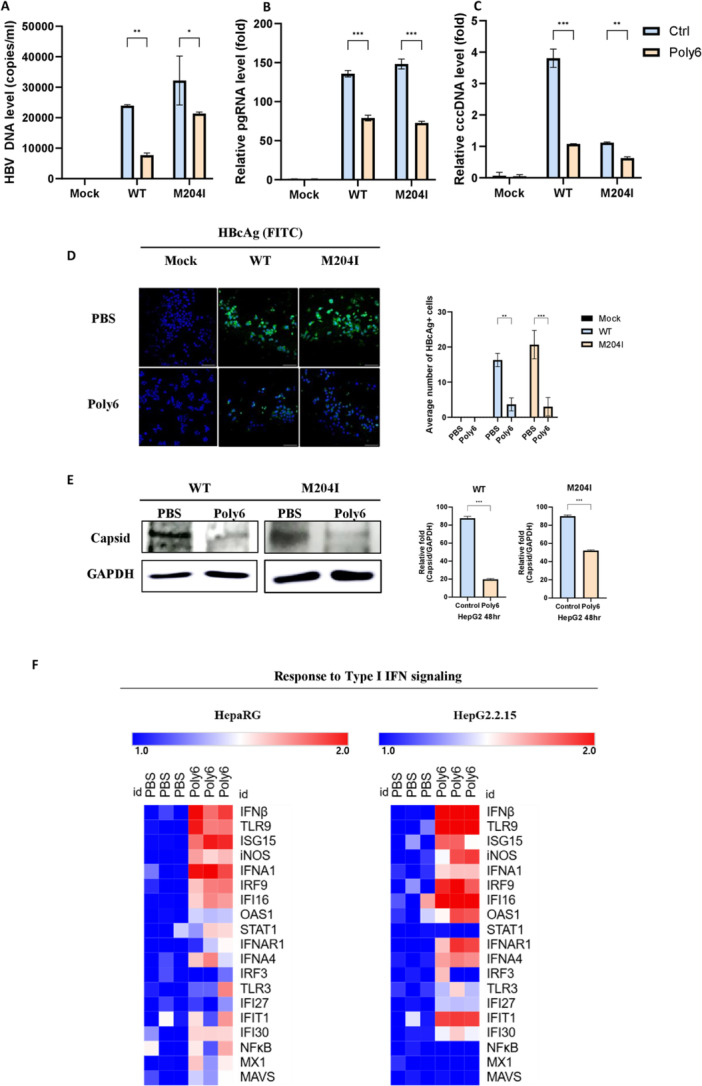
Antiviral effects of Poly6 against drug‐resistant HBV variants. (A–B) HBV DNA and pgRNA levels in HepG2 cells transfected with 1.2× WT or 1.2× M204I plasmids and treated with PBS or Poly6 (10 μM). (C) cccDNA levels in HepG2 cells transfected with linearized 1.2× WT or M204I plasmids after drug treatment. (D–E) HBcAg expression (Alexa 594) and capsid formation were assessed by immunofluorescence and western blot using SDS–PAGE and nonreducing native gels in HepG2 cells treated with PBS or Poly6, and quantification of HBcAg was performed from multiple randomly acquired confocal fields to avoid selection bias. (F) Quantitative PCR array analysis examining the expression of IFN‐I response genes in HBV‐infected primary HepaRG cells and HepG2‐2.15 cells.

A significant limitation of nucleos(t)ide analog therapy is an increased risk of drug resistance [31]. The rtM204I mutation in the YMDD motif of the pol gene is one of the most well‐known resistance variants [23, 24]. To evaluate Poly6 susceptibility, we performed drug response assays using both WT and M204I mutant vectors. After transfection of the Mock‐, WT‐, and M204I‐containing vectors into HepG2 cells, HBV DNA, pgRNA, and cccDNA were measured by RT–qPCR. Consequently, HBV DNA, pgRNA, and cccDNA levels were reduced in the Poly6 group compared to those in the control group in both WT‐ and M204I‐transfected cells (Figure 3A–C). The levels of HBV core protein and capsid formation were analyzed using immunofluorescence and Western blot assays. As shown in Figure 3D–E, Poly6 potently inhibited HBcAg and capsid formation in both WT‐ and M204I‐transfected cells. Taken together, WT and the M204I mutant show susceptibility to Poly6.

### 3.5 The Antiviral Activity of Poly6 Against HBV Depends on IFN‐I Signaling

**Figure 4:**
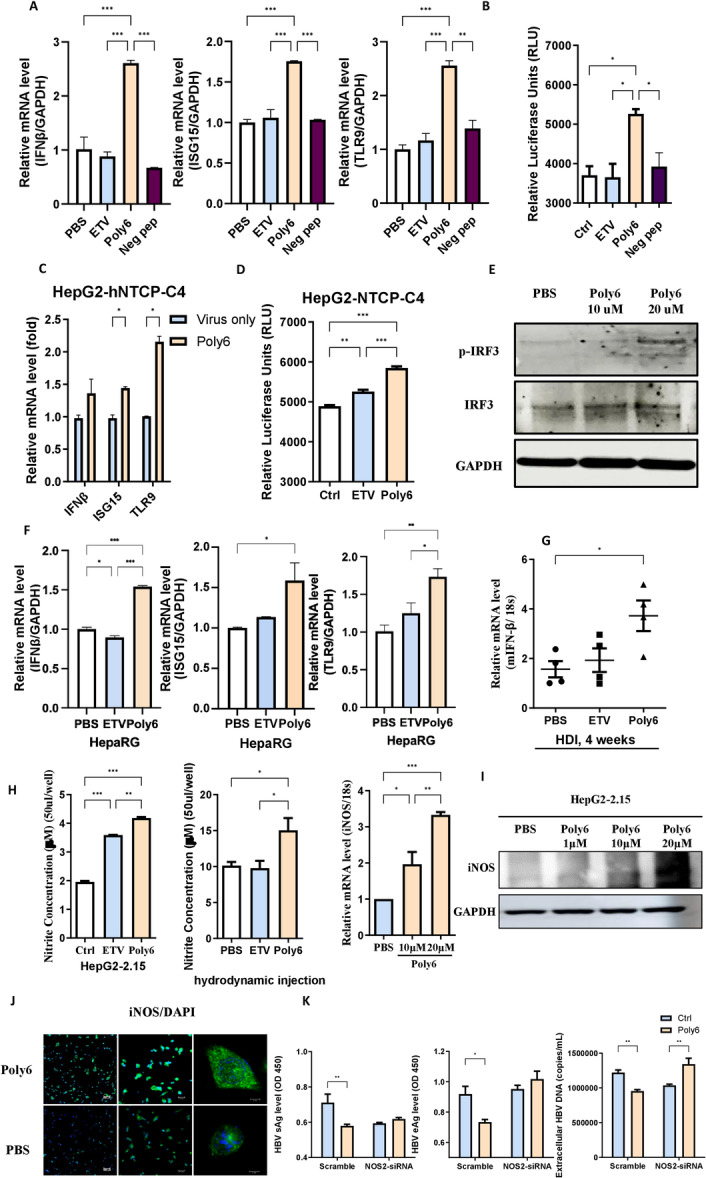
Anti‐HBV activity of Poly6 via the IFN‐I pathway. (A) RT–qPCR analysis of IFN‐β, ISG15, and TLR9 mRNA in HepG2.2.15 cells. (B) IFN‐I secretion measured in hMH55‐293‐IFN‐β‐ISRE cells at different time points after treatment. (C) IFN‐β, ISG15, and TLR9 mRNA levels in HepG2‐NTCP‐C4 cells treated with PBS or Poly6 (10 μM). (D) IFN‐I expression in hMH55‐293‐IFN‐β‐ISRE cells following treatment with Poly6 or ETV. (E) IRF3 and phospho‐IRF3 protein levels in HepG2‐NTCP‐C4 cells after PBS or Poly6 treatment. (F) RT–qPCR analysis of IFN‐β, ISG15, and TLR9 mRNA in HepaRG cells. (G) IFN‐β mRNA levels in liver tissues of HBV hydrodynamic injection mouse model. (H) Nitrite concentration (left) in supernatants of HepG2.2.15 cells or serum from hydrodynamic mice treated with PBS, ETV (30 nM), or Poly6 (10 μM), and iNOS mRNA levels (right) measured by RT–qPCR. (I) iNOS protein expression detected by western blot. (J) Confocal images of iNOS in HepG2.2.15 cells treated with PBS or Poly6. (K) Treatment of iNOS‐siRNA to confirm the iNOS‐dependent antiviral effects of Poly6. Data are mean ± SD of three independent experiments with 95% CI. *p < 0.05, **p < 0.01, ***p < 0.001 versus PBS.

To investigate the molecular mechanisms underlying the inhibitory effects of Poly6 on HBV cccDNA stability, pgRNA expression, and capsid formation, we carried out quantitative polymerase chain reaction (qPCR) array analysis in in HBV‐infected primary HepaRG cells and HepG2‐2.15 cells, enabling comprehensive profiling of host transcriptional changes [32]. Targeted gene expression profiling demonstrated increased expression of IFN‐I signaling–associated genes (Figure 3F), including marked upregulation of IFN‐β, OAS1, ISG15, and multiple additional interferon‐stimulated genes (ISGs) which were further validated by RT‐qPCR with negative peptide (Figure 4A). These findings build upon our previous observations that Poly6 functions as an IFN‐I inducer in other viral and immunotherapeutic contexts [21, 33], thereby underscoring IFN‐I pathway activation as the principal mechanistic axis of Poly6‐mediated HBV inhibition. We then assessed the IFN‐I secretion with hMH55‐293‐ISRE cells, which reflects luminescence reaction based on the IFN‐I level [34]. Poly6 markedly enhanced the luminescence signal, while the negative peptide showed no detectable induction (Figure 4B). Consistently, Poly6 upregulated IFN‐β, ISG15, and TLR9 expression, enhanced IFN‐induced luminescence, and increased phospho‐IRF3 levels in the HepG2‐hNTCP‐C4 infection model (Figure 4C–E). Moderate increased expression of the IFN‐I‐related genes was also observed in HepaRG cells, further supporting the immunomodulatory activity of Poly6 in hepatocyte systems (Figure 4F). Moreover, intravenous Poly6 treatment elevated IFN‐β mRNA in liver tissues of hydrodynamic injection mouse models compared with controls (Figure 4G).

### 3.6 Poly6‐induces iNOS Upregulation and NO‐Dependent Inhibition of HBV Replication

qPCR array profiling revealed that Inducible Nitric Oxide Synthase (iNOS/NOS2) was upregulated within the set of ISGs upon Poly6 treatment in HBV‐infected hepatocytes. To verify its potential contribution to the Poly6‐mediated antiviral activity, we first examined iNOS induction and its functional relevance in HBV models. Poly6 markedly increased nitrite levels in treated cells as well as in liver tissues of HDI mice as measured by the Griess reagent system (Figure 4H). Consistently, Poly6 strongly upregulated iNOS mRNA and protein expression in a dose‐dependent manner, as confirmed by RT‐qPCR, Western blotting, and confocal microscopy (Figure 4I–J). To assess the functional relevance of iNOS induction, we performed iNOS knockdown using siRNA. Suppression of iNOS expression abolished the antiviral activity of Poly6, indicating that the Poly6‐mediated inhibition of HBV replication is iNOS dependent (Figure 4K). These findings indicate that Poly6‐driven iNOS upregulation is essential for its antiviral activity and support a model in which Poly6 stimulates NO production, leading to nitric‐oxide–dependent disruption of HBV nucleocapsid assembly [35, 36, 37].

### 3.7 Neutralizing the IFN‐I Receptor Abrogates the Anti‐HBV Activity of Poly6

**Figure 5:**
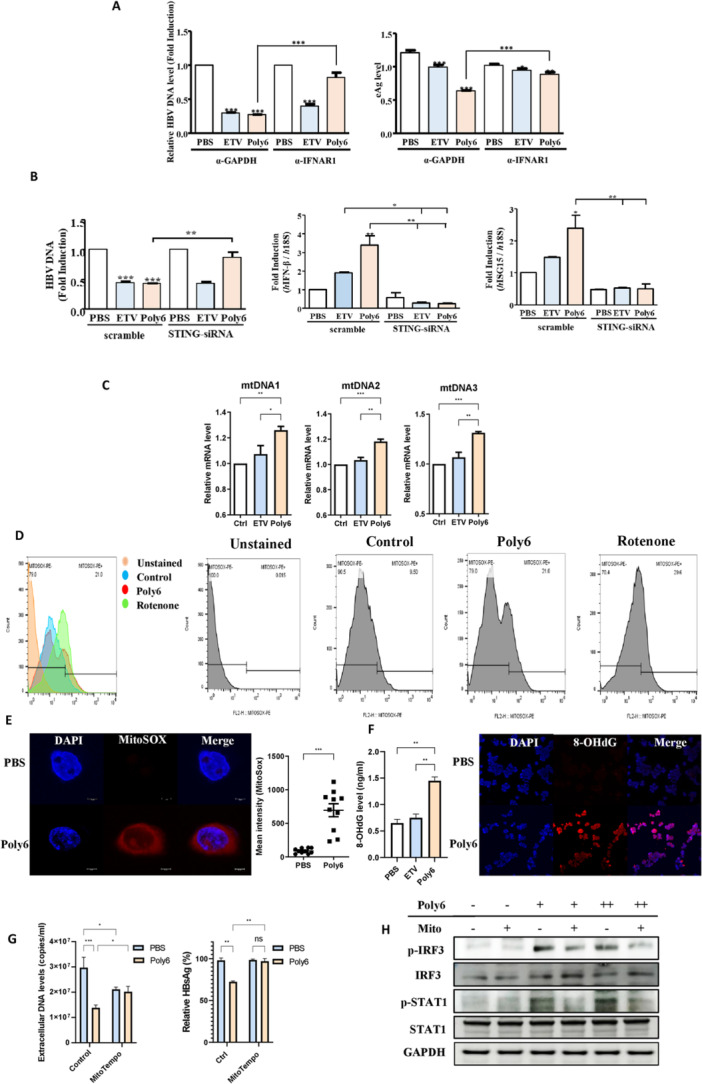
The antiviral activity of Poly6 against HBV depends on IFN‐I induction and Poly6 induces mitochondrial DNA stress via mtROS production (A) HBV DNA and HBeAg levels in HepG2.2.15 cells treated with PBS, ETV (30 nM), or Poly6 (10 μM), followed by incubation with anti‐GAPDH or anti‐IFNAR1 (2.5 μg/ml). (B) Extracellular HBV DNA and IFN‐β, ISG15 mRNA levels in HepG2.2.15 cells transfected with STING siRNA and treated with Poly6. (C) Cytosolic mtDNA quantified by RT–qPCR in Poly6‐treated HepG2.2.15 cells. (D) Flow cytometry histograms showing mitochondrial ROS accumulation after 12 h of Poly6 treatment. (E) Confocal images of mitochondrial superoxide in Poly6‐treated HepG2.2.15 cells. (F) 8‐OHdG ELISA and immunofluorescence in HepG2.2.15 cells treated with PBS, ETV (30 nM), or Poly6 (10 μM). (G) Antiviral effect of Poly6 in the presence of MitoTEMPO, HBV DNA and HBsAg levels were measured. (H) Protein expression in HepG2.2.15 cells treated with PBS or Poly6 (5 or 10 μM), with or without MitoTEMPO. Data are mean ± SD of three independent experiments with 95% CI. *p < 0.05, **p < 0.01, ***p < 0.001.

To confirm the type‐I‐IFN‐dependent antiviral effect of Poly6 against HBV, we performed a neutralizing assay. HepG2.2.15 cells were preincubated with a control antibody (GAPDH) or a neutralizing antibody (α‐IFNAR1) to block the activity of the IFN receptor and were then treated with PBS, ETV, or Poly6. After 2 days, the IFN receptor neutralizing antibody (α‐IFNAR1) abrogated the anti‐HBV effects of Poly6. We observed that the diminished HBV viral DNA and HBeAg levels caused by Poly6 in the control group were not found in the IFNAR1 neutralizing group (Figure 5A). These results indicate that IFN‐1 signaling is essential for the antiviral activity of Poly6.

### 3.8 The Antiviral Activity of Poly6 Against HBV Depends on IFN‐I Induction via the STING/IRF3 Pathway

Two well‐known upstream pathways of IFN‐I secretion associated with HBV infection: cGAS/STING [38] and RIG‐I/MAVS [39] signaling. DNA viral infection is known to especially activate cGAS/STING signaling, which causes phosphorylation of IFN regulatory factors (IRFs) and gives rise to induction of IFN‐I [32]. In our experiments, Poly6 increased RIG‐I mRNA level in HepG2 cells (Supporting Information Figure S6A), but it still had strong anti‐HBV effects in Huh7.5 cells, which have a point mutation that disrupts RIG‐1 signaling (Supporting Information Figure S6B) [40]. Therefore, we hypothesized that Poly6‐induced IFN‐I is mainly mediated by the cGAS/STING pathway. To test this, we used STING siRNA, which reversed the Poly6‐dependent reduction of HBV DNA and suppressed the induction of IFN‐β and ISG15 mRNA levels (Figure 5B). These data suggest that Poly6‐induced IFN‐I is downstream of cGAS/STING signaling.

### 3.9 Mitochondrial ROS and Oxidized DNA Release Induced by Poly6 Promote IFN‐I Production

It has been reported that oxidized DNA released due to mitochondrial stress contributes to IFN‐I induction, particularly via the cGAS/STING signaling [41, 42, 43]. We examined whether Poly6 induces IFN‐I through mitochondrial stress‐dependent manner. Cytoplasmic mtROS and three mtDNA regions were analyzed. As shown in Figure 5C, Poly6 progressively elevated cytosolic mtDNA levels, indicating mtDNA release into the cytoplasm. Next, to investigate mtROS production induced by Poly6, we measured mitochondrial superoxide using MitoSOX. FACS analysis revealed a clear histogram shift in the Poly6‐treated group in a dose‐dependent manner (Figure 5D and Supporting Information Figure S7). Similarly, our confocal imaging also showed a clear increase in mtROS in the Poly6 group (Figure 5E and Supporting Information Figure S8). Meanwhile, mitochondrial ROS can induce oxidative mtDNA damage, and 8‐OHdG is a useful biomarker indicating oxidative stress [44]. 8‐OHdG were higher in the Poly6‐treated group (Figure 5F). To further test whether the antiviral and IFN‐I–inducing effects of Poly6 depend on mitochondrial stress, we used Mito‐TEMPO, a mitochondrial superoxide scavenger. Mito‐TEMPO reversed the Poly6‐induced reduction of HBV DNA and HBsAg, and suppressed IFN‐I signaling activation (Figure 5G–H). Taken together, these results indicate that mitochondrial ROS and oxidized mtDNA release are key mediators of Poly6‐induced IFN‐I signaling and its anti‐HBV activity.

### 3.10 Poly6 Strongly Induces IFI16 Expression and Enhances Its Binding Affinity to Sp1

**Figure 6:**
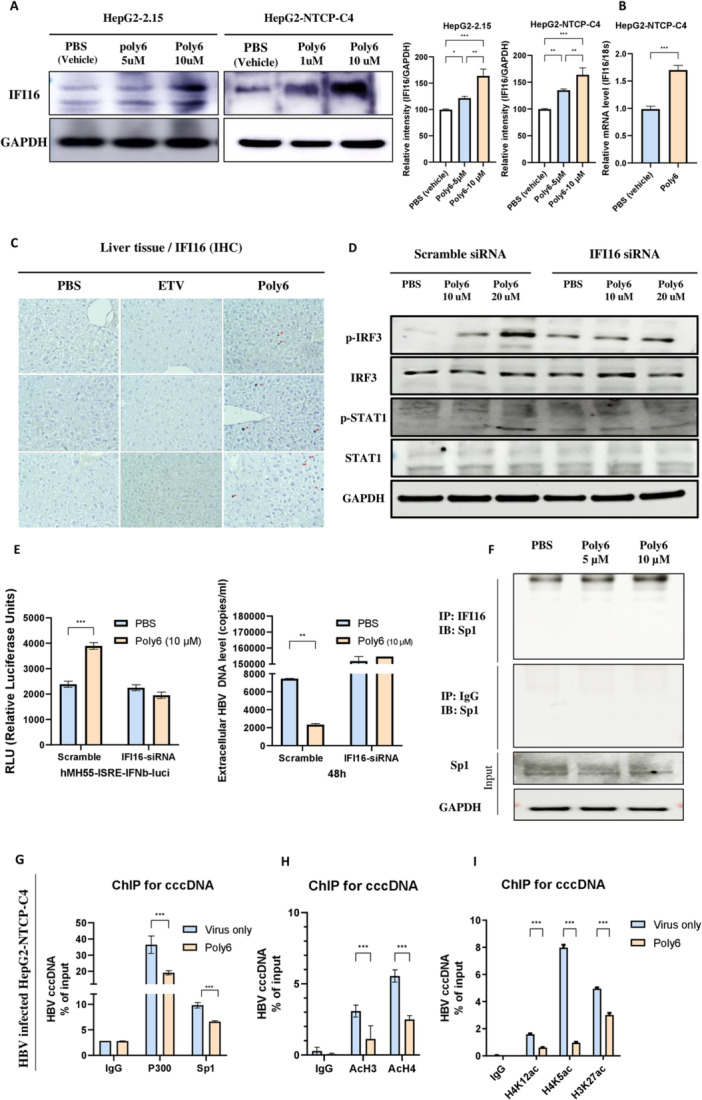
Poly6 promotes IFI16 expression and Sp1 binding, contributing to cccDNA epigenetic regulation. (A) IFI16 expression in HepG2.2.15 and HepG2‐NTCP‐C4 cells analyzed by western blot, relative intensity quantified. (B) IFI16 mRNA levels in HepG2‐NTCP‐C4 cells treated with PBS or Poly6. (C) IFI16 expression in liver tissues detected by immunohistochemistry. (D) Western blot analysis of IRF3, p‐IRF3, STAT1, p‐STAT1, and GAPDH in HepG2‐NTCP‐C4 cells after IFI16 siRNA transfection, HBV infection, and treatment with PBS or Poly6. (E) Secreted IFN‐I and extracellular viral DNA in HBV‐infected HepG2‐NTCP‐C4 cells following IFI16 knockdown and treatment with PBS or Poly6. (F) Immunoprecipitation of IFI16 or IgG control from Poly6‐ or PBS‐treated HepG2.2.15 cells, bound proteins detected by anti‐Sp1 and anti‐GAPDH. (G–I) ChIP assays showing recruitment of p300 and Sp1 (G), AcH3 and AcH4 (H), and H3K27ac, H4K12ac, and H4K5ac (I) to cccDNA in HepG2‐NTCP‐C4 cells 4 days post‐infection.

Building on the observation that Poly6 induces IFN‐I responses and downstream ISGs, we next focused on IFI16 as an additional effector within this cascade. Given its role as a nuclear DNA sensor implicated in cccDNA regulation, we examined whether Poly6‐induced IFI16 contributes to epigenetic suppression of viral transcription [19, 45]. Our qPCR array data indicated a significant induction of IFI16 in Poly6‐treated hepatocytes (Figure 3F), and Poly6 increased IFI16 protein levels in a dose‐dependent manner in HepG2‐NTCP‐C4 and HepG2.2.15 cells (Figure 6A). Consistently, a quantitative analysis confirmed a robust upregulation of IFI16 mRNA in HBV‐infected HepG2‐NTCP‐C4 cells (Figure 6B). Liver immunohistochemistry (IHC) showed that treatment with Poly6 led to notable increases in the number of IFI16‐positive hepatocytes (Figure 6C and Supporting Information Figure S9). Then, to investigate whether the Poly6‐induced IFN‐I is mediated by IFI16, we performed IFI16 knockdown. As shown in Figure 6D–E, IFI16‐siRNA abolished Poly6‐induced IFN‐I production and the antiviral activity, suggesting that IFI16 mediates Poly6‐driven signaling through the STING/IRF3 axis. Meanwhile, as IFI16 is known to suppress virus transcription by interfering with Sp1‐dependent gene expression [46], we tested the effect of Poly6‐induced IFI16 on the Sp1 binding. Immunoprecipitation assays revealed that Sp1 was pulled down with IFI16, but not with normal IgG control, supporting a dose‐dependent interaction between IFI16 and Sp1. (Figure 6F).

### 3.11 Poly6‐induced IFI16 Epigenetically Suppressed HBV cccDNA

Next, we demonstrated the effect of poly6‐induced IFI16 on minichromosomes of cccDNA in HepG2‐NTCP‐C4 cells. ChIP assay analysis showed that Poly6 impaired the recruitment of the acetyltransferase p300 as well as Sp1 to cccDNA (Figure 6G). Furthermore, we examined the effect of Poly6 on epigenetic regulation in cccDNA. Poly6 treatment reduced the H3 and H4 acetylation levels, particularly H3K27ac, H4K5ac, and H4K12ac on cccDNA minichromosomes (Figure 6H–I). Considering our results together, Poly6 epigenetically suppressed the HBV cccDNA through the IFI16/Sp1 axis.

## 4 Discussion

In this study, we show that Poly6 suppresses HBV replication through a coordinated mechanism centered on IFN‐I signaling. A key observation was that Poly6 activated two distinct nucleic acid–sensing routes: mitochondrial stress that caused the release of oxidized mtDNA into the cytosol, and nuclear sensing mediated by IFI16 [47]. These events converged on the STING–IRF3 pathway and led to a sustained IFN‐I response. The resulting antiviral state affected HBV at multiple levels, reducing pgRNA transcription, destabilizing nucleocapsids, and imposing epigenetic repression of cccDNA. Together, these findings place Poly6 within a class of host‐directed antiviral molecules that modulate intrinsic defense pathways.

The downstream consequences of Poly6‐induced interferon signaling were multifaceted. One arm involved robust induction of iNOS, which generated nitric oxide and interfered with nucleocapsid assembly, consistent with previous reports describing NO‐mediated inhibition of HBV replication. In parallel, Poly6 markedly increased IFI16 expression. Beyond functioning as a DNA sensor that contributes to interferon induction, IFI16 acted as a transcriptional repressor by limiting Sp1 access to cccDNA and reducing histone acetylation at several sites. These complementary activities—iNOS‐dependent capsid disruption and IFI16‐driven chromatin remodeling—illustrate how Poly6 amplifies endogenous antiviral programs to limit HBV replication.

The mechanistic features of Poly6 differ in several important respects from conventional IFN‐α therapy. IFN‐α administration exposes multiple tissues to high systemic interferon levels and is frequently limited by dose‐dependent adverse effects [48]. In contrast, Poly6 preferentially enhanced IFN‐I responses in hepatocytes, the primary site of viral persistence, suggesting a more localized and potentially better‐tolerated mode of action. Poly6 also engages pathways not fully utilized by exogenous interferon, including mitochondrial DNA stress and IFI16‐mediated chromatin regulation, resulting in broader antiviral coverage than cytokine supplementation alone. These distinctions may explain why Poly6 suppressed both HBV DNA and HBsAg in vivo, with effects that were in some cases more pronounced than those observed with nucleos(t)ide analogs.

**Figure 7:**
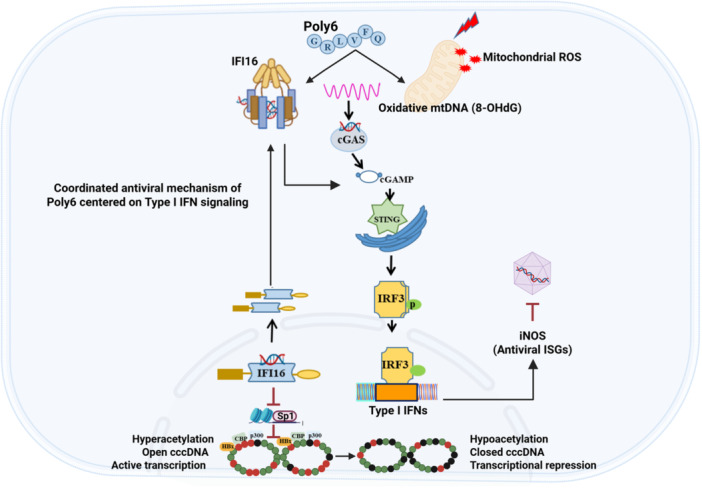
Proposed model of Poly6 activity. Poly6 restricts HBV replication by triggering IFI16‐ and mtDNA‐dependent sensing pathways that activate STING–IRF3 and sustain IFN‐I signaling, leading to reduced HBV transcription, iNOS‐mediated capsid disruption, and IFI16‐driven epigenetic silencing of cccDNA.

Another notable aspect of Poly6 is its activity against the NA‐resistant M204I variant (Figure 3A–E). As drug resistance remains a major limitation of current antiviral therapy, the susceptibility of this variant to Poly6 suggests its potential utility in treatment settings where existing drugs are ineffective. In addition, Poly6 showed synergy with entecavir in combination assays (Figure 1L), raising the possibility that it could be incorporated into therapeutic regimens that pair direct‐acting antivirals with host‐targeted agents to increase depth and durability of viral suppression (Figure 7)

Poly6 may also have broader immunological implications. Previous studies have demonstrated that Poly6 enhances antigen‐specific immune responses through IFN‐I–dependent activation of dendritic cells [33, 49]. When viewed together with the present findings, these observations suggest that Poly6 could exert dual benefits in chronic HBV infection by directly suppressing viral replication and simultaneously supporting an immune restoration approach that aligns with emerging strategies aimed at achieving a functional cure.

This study has limitations. Although HDI and TG mouse models capture important features of HBV replication, they do not fully recapitulate de novo cccDNA formation in human hepatocytes. Validation in specialized systems that allow physiologic establishment and maintenance of cccDNA will be important for future studies. Furthermore, pharmacokinetic, toxicity, and biodistribution profiles have not yet been defined and will be essential for assessing clinical feasibility.

In summary, Poly6 exerts multilayered antiviral effects by repressing cccDNA transcription via activating endogenous IFN‐I pathways, which is involved mainly two pathways including IFI16‐mediated chromatin modification and iNOS‐dependent capsid disruption. Poly6 also demonstrated activity against drug‐resistant variants, synergy with approved antivirals, and the ability to potentiate immune responses, making it a promising candidate for future therapeutic strategies toward durable control and potential functional cure of chronic HBV infection (Schematic Abstract and Figure. S10).

## Author Contributions

**Junghwa Jang:** writing, methodology, investigation, data curation. **Dong Hyun Kim:** methodology, investigation. **Ziyun Kim:** methodology, investigation. **Eunseo Kim:** methodology, investigation. **Yu‐Min Choi:** writing – original draft, methodology, investigation, formal analysis, data curation. **Bum‐Joon Kim:** writing – original draft, methodology, investigation, formal analysis, data curation, funding acquisition.

## Ethics Statement

The animal study was approved by the Seoul National University Institutional Animal Care and Use Committee (IACUC) [SNU‐180530‐1‐2, SNU‐230103‐5‐2, and SNU‐230622‐1‐1].

## Conflicts of Interest

The authors declare no conflicts of interest.

## Supporting information

Supplemental_files_REVISION_2.

## Data Availability

The data that support the findings of this study are available on request from the corresponding author. The data are not publicly available due to privacy or ethical restrictions.
