# Supplementary material for: An HBV‐Derived Peptide Poly6 as a Novel Candidate for Functional Cure Via IFN‐I–Mediated Epigenetic Regulation of cccDNA
Source: J Med Virol. 2026 Mar 27;98(4):e70877. doi: 10.1002/jmv.70877 (PMC13023030; doi:10.1002/jmv.70877)
Supplement: Supplementary file 1 — Supplemental_files_REVISION_2. [file JMV-98-e70877-s001.pdf]

An HBV-Derived Peptide Poly6 as a Novel Candidate for Functional Cure via IFN-I–Mediated Epigenetic Regulation of cccDNA

Junghwa Jang, Dong Hyun Kim, Ziyun Kim, Eunseo Kim, Yu-Min Choi and Bum-Joon Kim

Table of contents

Supplementary materials and methods……………………………………………………2

List of primers……………………………………………………………………………..6

Supplementary figures……………………………………………………………… ……7

List of Abbreviations…………………………………………………………………...18

Raw Ct Table……………………………………………………………...…………..…19

Western blot – raw data…………………………………………………………...……20

Supporting Materials

Supplementary material and methods

1. Reagents and antibodies

Antibodies against HBc (B0586) were purchased from Dako (Agilent Tech., Santa Clara, CA), and anti-HSP90 (#4877), STAT1 (#9172), IRF3 (#4302S) and p-IRF3 (#29047S) were purchased from Cell Signaling (Cell Signaling, Danvers, MA). Antibodies against GAPDH (sc25778) and p-STAT1 (sc7988) were obtained from Santa Cruz Biotechnology (Santa Cruz, Dallas, TX). IFNAR1 (ab10739) and LaminB1 (ab16048) antibodies were purchased from Abcam (Abcam, UK). Carboxy-H_2DCFDA (C400, ThermoFisher, CA) for indicating ER stress and Mito-SOX (M36008, Molecular Probes, UK) for staining mitochondrial superoxide were used in this study. STING siRNA (sc-92042) was obtained from Santa Cruz Biotechnology (Santa Cruz, Dallas, TX).

2. Cell Viability Assay.

HepG2, HepG2.2.15 or HepG2-hNTCP-C4 cells were plated (1 x 10^4 cells/well) in 96-well microplates and treated with ETV or Poly6 for 5 days. For the MTS assay, Cell-Titer 96 Aqueous One Solution (Promega, London, UK) was treated directly to wells, and the cells were incubated for 3 h. The plates were read at 490 nm absorbance.

3. HBsAg, HBeAg and 8-OHdG ELISAs.

To measure the secreted HBsAg and HBeAg from the culture supernatants, HBsAg and HBeAg ELISAs were carried out according to the provided experimental protocols. For the measurement of 8-OHdG production, 8-OHdG analysis ELISA system (Cell Biolabs, CA) was applicated following the manufacturer's protocol.

4. Detection of HBV DNA.

The cell pallets were harvested, and HBV DNA was collected using QIAamp DNA Mini Kit (QIAGEN, Hilden, Germany). HBV DNA was analyzed using qPCR with SYBR.

5. HBV cccDNA Assay.

HBV cccDNA was isolated as described previously (1). Briefly, HepG2.2.15, HepG2, and HepG2-NTCP-C4 cells were lysed in cytosolic lysis buffer. After collecting the cytosolic lysates, the nuclear pellets were sonicated in nuclear lysis buffer and incubated overnight at 37 °C with proteinase K. The resulting extracts were purified by phenol–chloroform extraction (1:1). To ensure that only cccDNA was measured, all non-cccDNA forms, including plasmid DNA and replicative intermediates, were removed by treatment with Plasmid Safe ATP-dependent DNase (PSAD). To compare digestion efficiency, cccDNA was examined following PSAD treatment, and in parallel, a subset of samples was further analyzed after DpnI/T5 exonuclease digestion to more stringently remove plasmid/rcDNA. Real-time-qPCR was carried out with specific primers. For detection of cccDNA by electrophoresis method, HBV cccDNA was amplified with cccDNA-selective primers [forward, 50-GGGGCGCACCTCTCTTTA-30 (position 1523 to 1540); reverse, 50-AGGCACAGCTTGGAGGC-30 (position 1886 to 1870)] and cccDNA- nonselective primer [forward, 50-CACTCTATGGAAGGCGGGTA-30 (position 2755 to 2774); reverse, 50-TGCTCCAGCTCCTACCTTGT-30 (position 3002-2983)] Amplified products were then detected by 1% agarose gel electrophoresis. cccDNA was quantified by qPCR following DNase digestion and normalized to total DNA input.

6. Measurement of reactive oxygen species (ROS).

Cells were treated with 5 μM carboxy-H2DCFDA (D399, ThermoFisher, MA) or 5 nM MitoSox (M36008, Molecular Probes, UK). For flow cytometry, the cell pellets were resuspended in FACS buffer and fixed with 4% PFA. To protect against mitochondrial dysfunction, 10 μM MitoTempo (Sigma Aldrich, MO) was pretreated for 12 h in this study, and 0.5 μM rotenone (Sigma Aldrich, MO) was used as a positive control for inducing ROS via mitochondrial stress. Cytosolic mitochondrial DNA (mtDNA) was prepared using a Mitochondrial DNA Isolation kit (ab65321, Abcam, UK), and RT-qPCR was conducted to quantitate the level of mtDNA using specific primers.

7. Confocal for Detection of mitochondrial ROS (mtROS)

Cells seeded in 2-channel glass slides (Nunc, Roskilde, Denmark) were stained with MitoSOX (1 μM). Nuclei were stained in mounting medium with DAPI (VECTASHIELD, H-1200).

8. Flow Cytometry for Detection of mitochondrial ROS

Cells were stained them with MitoSOX (5 μM) following the manufacturer's instruction. The mtROS levels were measured by the FACS Calibur system (BD Bioscience, San Jose, CA).

9. Type I Interferon Neutralization Assay.

For the neutralization assay, anti-IFNAR1 and anti-GAPDH antibodies were preincubated with cells for 2 h at RT with rotation, and the cells were seeded into plates. The next day, PBS, ETV or Poly6 were treated and incubated for 48 h.

10. Measurement of IFN-I Level Using *h*MH55-293-ISRE Cells

hMH55-293-ISRE cells were incubated with cell supernatant for 6-24 h and lysed with Reporter Lysis Buffer (E1500, Promega, Fitchburg, WI, United States). The lysate was treated

with Luciferase assay reagent (E1500, Promega, Fitchburg, WI, United States), and the reflective luminescence was analyzed using a TECAN (TECAN, Switzerland).

11. Knockdown of genes with siRNA

To knockdown the target gene, cells were transfected with siRNA. Cells were treated with OPTI-MEM containing siRNA (75 pmol) and Lipofectamine 3000 (7.5 µl) for 6 h.

12. Immunoprecipitation (IP)

Immunoprecipitation was carried out using Pierce Classic Magnetic IP/Co-IP Kit (Thermo Fisher Scientific, #88828) following the manufacturer's instruction.

13. qPCR array

Total mRNA was extracted from HepG2.2.15 cells, and the purity and quality of mRNA were measured according to the Minimum Information for Publication of Quantitative Real-Time PCR Experiments (MIQE) guidelines. Complementary DNA was synthesized using SensiFAST cDNA Synthesis Kit (Bioline). qPCR array was performed using AccuPower® qPCR Array (Bioneer, Daejeon, Korea). Data analysis was based on the $2^{-\Delta\Delta Ct}$ method. Average threshold cycles (C_t) for the gene of interest were obtained from triplicate samples and normalized by the C_t of HPRT as a housekeeping gene.

Supplementary Table S1. List of Primers.

Primer	Forward	Reverse
HBs	5'-TTG ACA AGA ATC CTC ACA ATA CC-3'	5' -GGA GGT TGG GGA CTG CGA AT- 3'
pgRNA	5'- GCC TTA GAG TCT CCG GAA CA-3'	5'- GAG GGA GTT CTT CTT CTA GG-3'
cccDNA	5' - CCG TGT GCA CTT CGC TTC A-3'	5'- GCA CAG CTT GGA GGC TTG A-3'
cccDNA selective primer	5'- GGG GCG CAC CTC TCT TTA -3'	5'- AGG CAC AGC TTG GAG GC-3'
cccDNA non-selective primer	5' -CAC TCT ATG GAA GGC GGG TA -3'	5'- TGC TCC AGC TCC TAC CTT GT -3'
Human 18S rRNA	5′- TAGAGGGACAAGTGGCGTTC -3'	5′- CGCTGAGCCAGTCAGTGT -3'
Human GAPDH	5'-CAC ATG GCC TCC AAG GAG TAA -3'	5'-GAG GGT CTC TCT CTT CCT CTT GT -3'

Supplemental Figure Legends

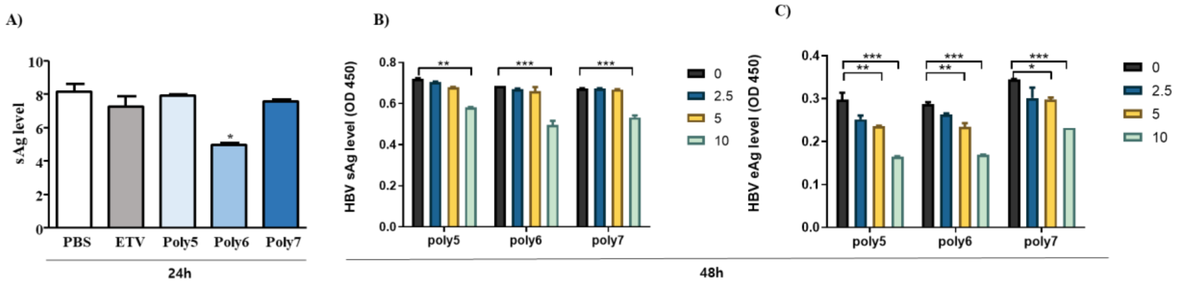

Supplementary Figure 1. Inhibition of HBV Replication by Three Candidates

HepG2.2.15 cells were treated with PBS, Entecavir (30nM), Poly5m Poly6, Poly7 (10μM) and incubated under standard culture conditions**.** (A) HBsAg level at 24 h and (B) HBsAg and (C) HBeAg levels at 48 h were measured using ELISAs. Each 0 μM (PBS, vehicle) bar corresponds to the internal control of its own independently performed experiment; thus, baseline values may differ slightly across panels. Data indicate the mean ± S.D. of three independently performed experiments. * $p < 0.05$, ** $p < 0.01$ and *** $p < 0.001$.

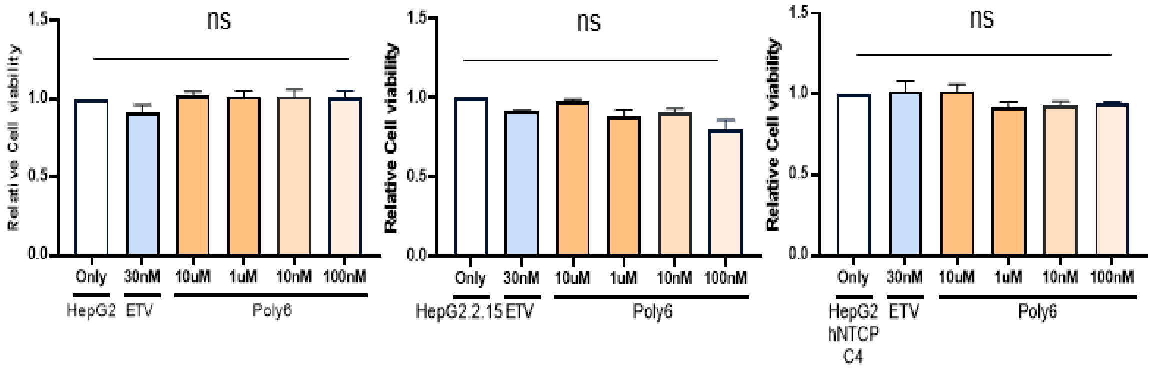

Supplementary Figure 2. Cell viability Cell viability of HepG2, HepG2.2.15, and HepG2-hNTCP-C4 cells treated with Poly6 for 3, 5, or 7 days, determined by MTS assay.

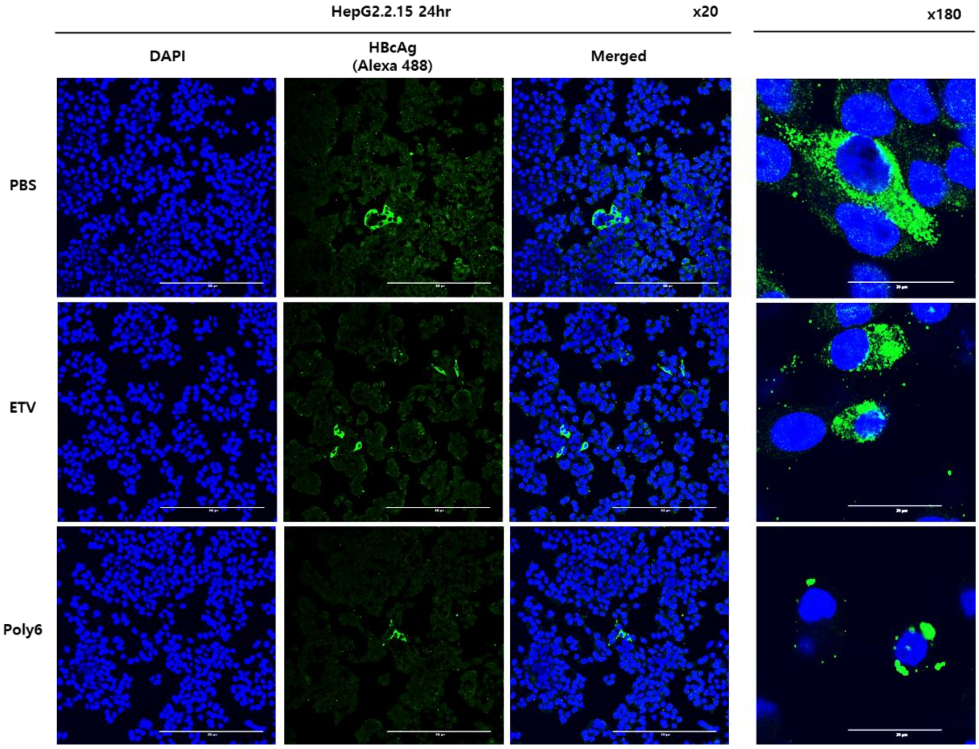

Supplementary Figure 3. Immunofluorescence of HBcAg in HepG2.2.15 cells. HepG2.2.15 cells were fixed and immunostained with an anti-HBcAg antibody (green) and counterstained with DAPI (blue). Confocal microscopy revealed HBcAg localization predominantly in the cytoplasm. Representative low- and high-magnification images are shown. Scale bars, 100 μm (left) and 10 μm (right).

A)

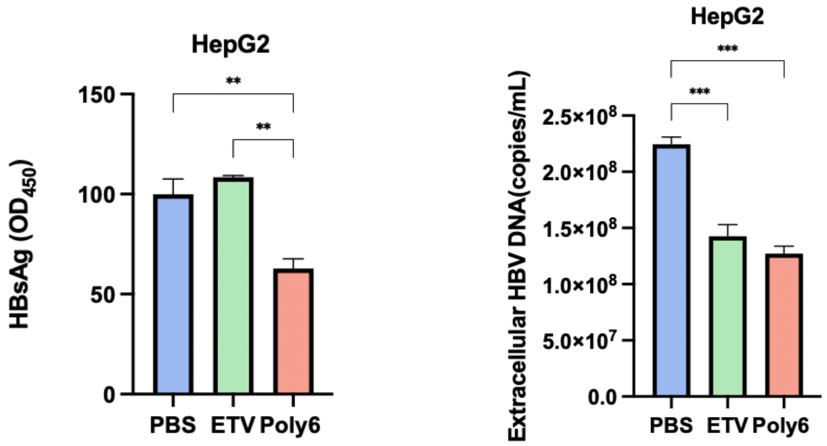

B)

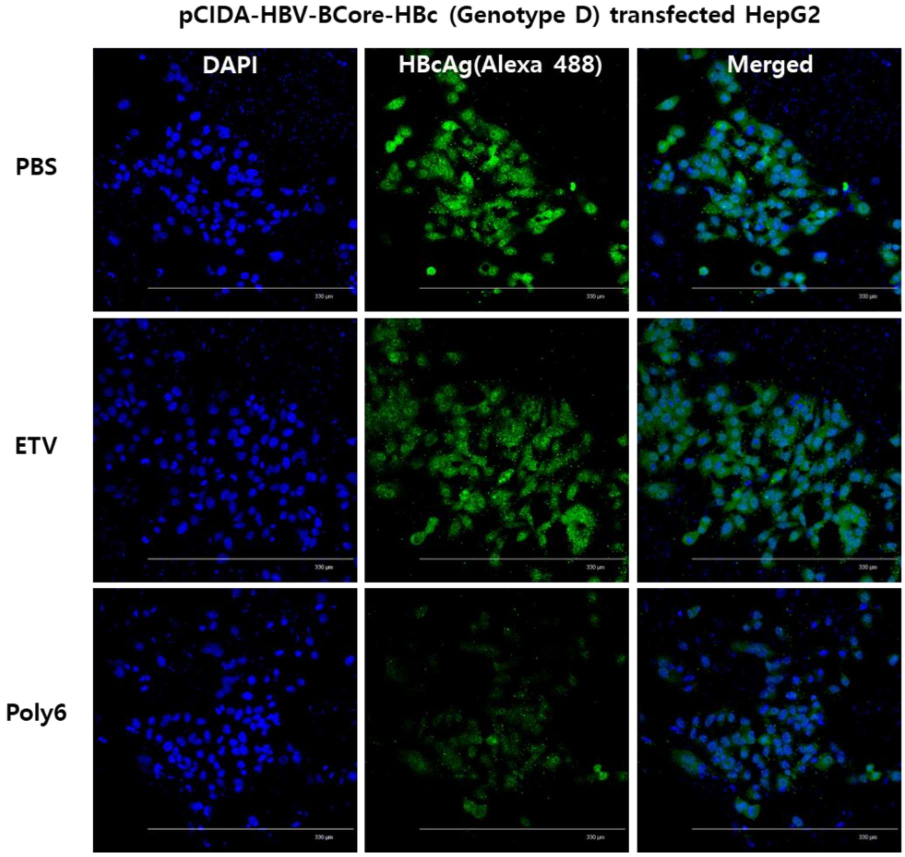

Supplementary Figure 4. Anti-HBV effect of Poly 6 in HBV genotype D

(A) HBsAg, HBV DNA level in HepG2 cells transfected with pCIDA-HBV-BCore-HBc (Genotype D) and treated with PBS, Entecavir or Poly6 for 48 hr. HBsAg and HBV DNA levels were analyzed by using ELISAs and qPCR, respectively. Data indicate the mean ± S.D. of three

independently performed experiments. ** $p < 0.002$, and *** $p < 0.001$ (B) Confocal images of HepG2 cells transfected with pCIDA-HBV(GenoD) and treated with PBS, Entecavir, or Poly6 for 48 hr. Cells were stained with Hep B cAg Alexa Fluor® 488(sc-23947) antibody. Scale bar = 100 μm

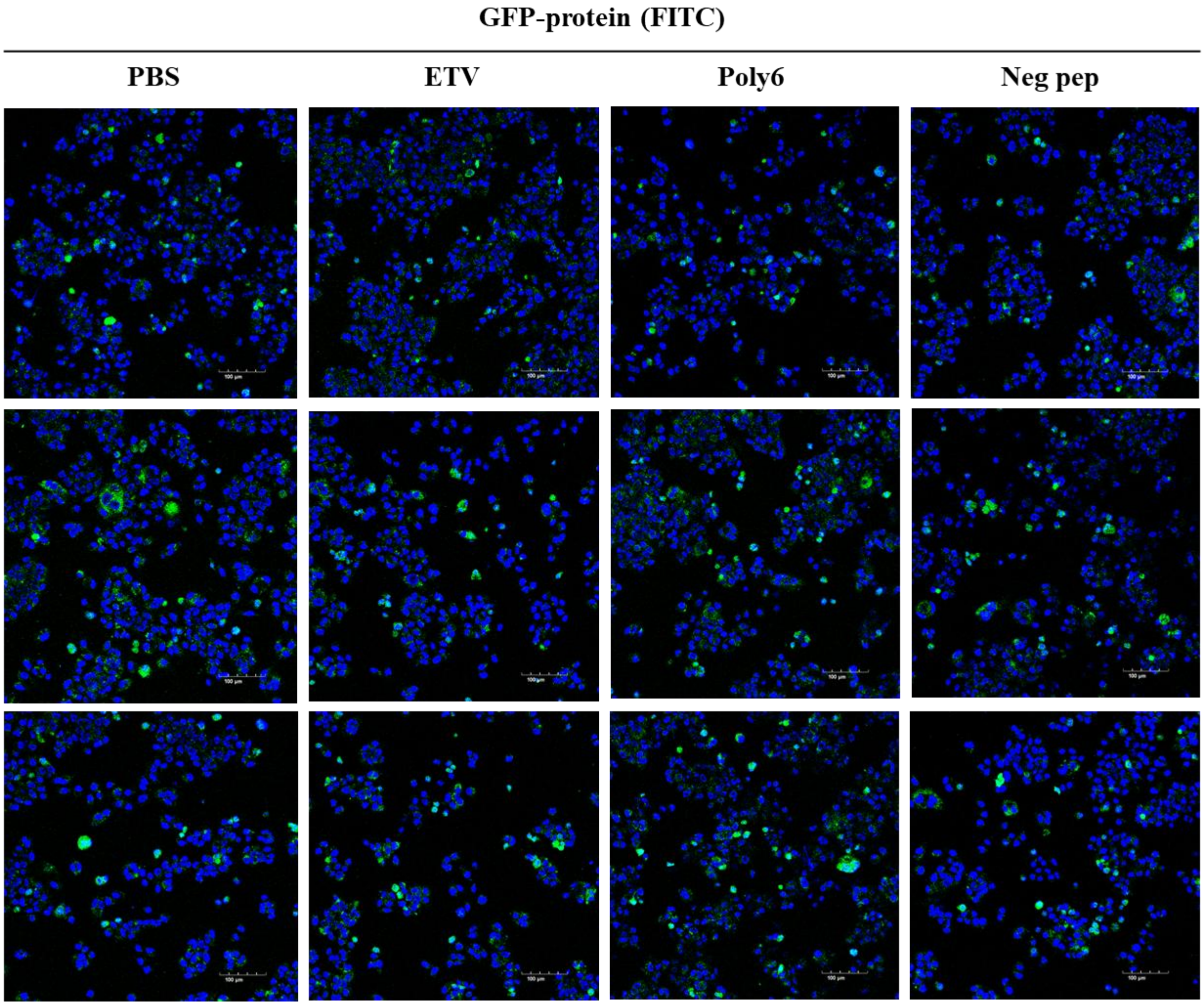

Supplementary Fig. 5. Poly6 does not affect GFP expression.

HepG2 cells transiently transfected with a GFP plasmid were treated with Poly6 (10 μM) or vehicle for 48 h. Confocal images show GFP (green) and nuclei (DAPI, blue). Poly6 did not alter GFP signal, indicating its inhibitory effect is specific to HBV proteins. Scale bar, 50 μm.

A)

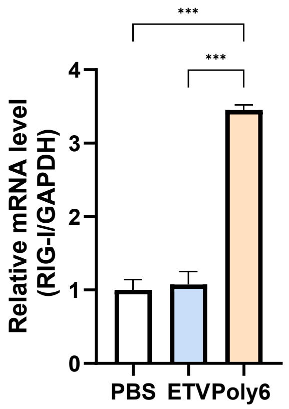

B)

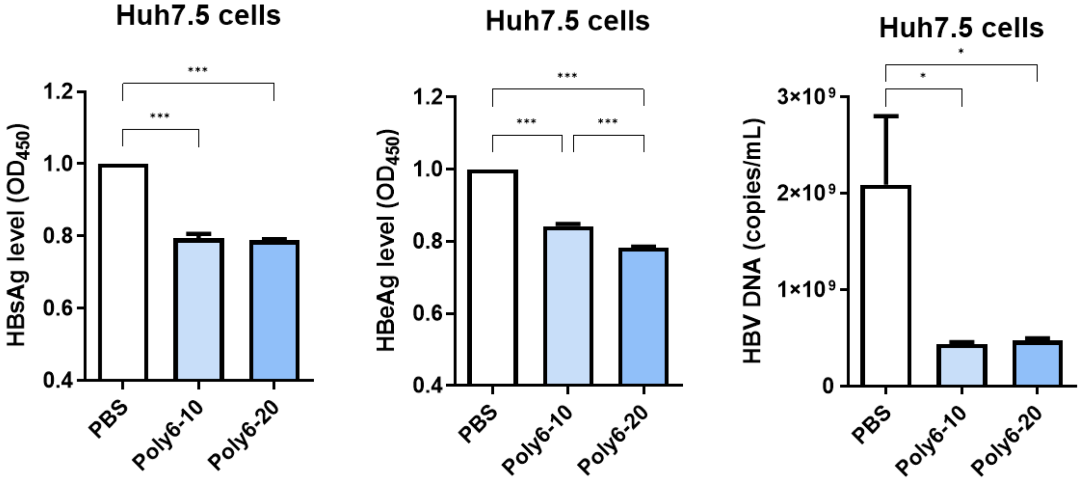

Supplementary Figure 6. Anti-HBV Activity of Poly6 in Huh7.5, the RIG-I-deficient Cells (A) RT–qPCR analysis of RIG-I mRNA in HepG2.2.15 cells. (B) HBsAg, HBeAg, and HBV DNA levels in the Huh7.5 cells transfected with 1.2x-WT were analyzed using ELISAs and qPCR. Data indicate the mean ± S.D. of three independently conducted experiments. * $p < 0.05$ and *** $p < 0.001$.

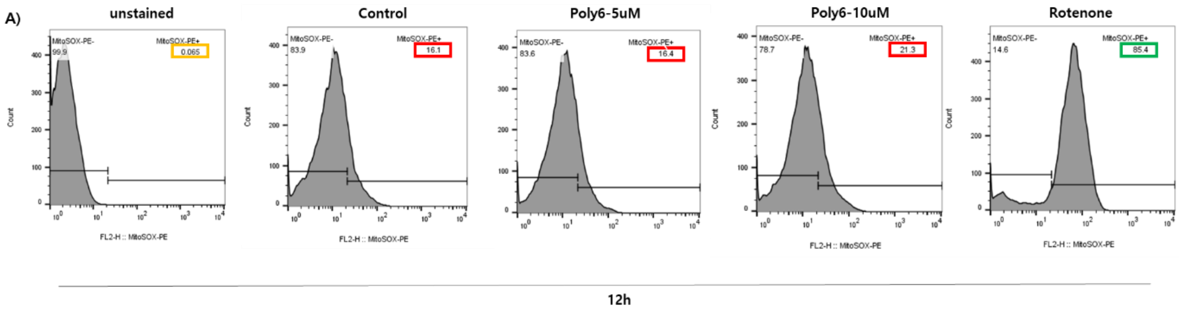

Supplementary Figure 7. Poly6 induces mtDNA Stress through Production of mtROS in Hepatocytes Representative histograms of flow cytometric assays showing shifted histograms following treatment with Poly6 for 12h..

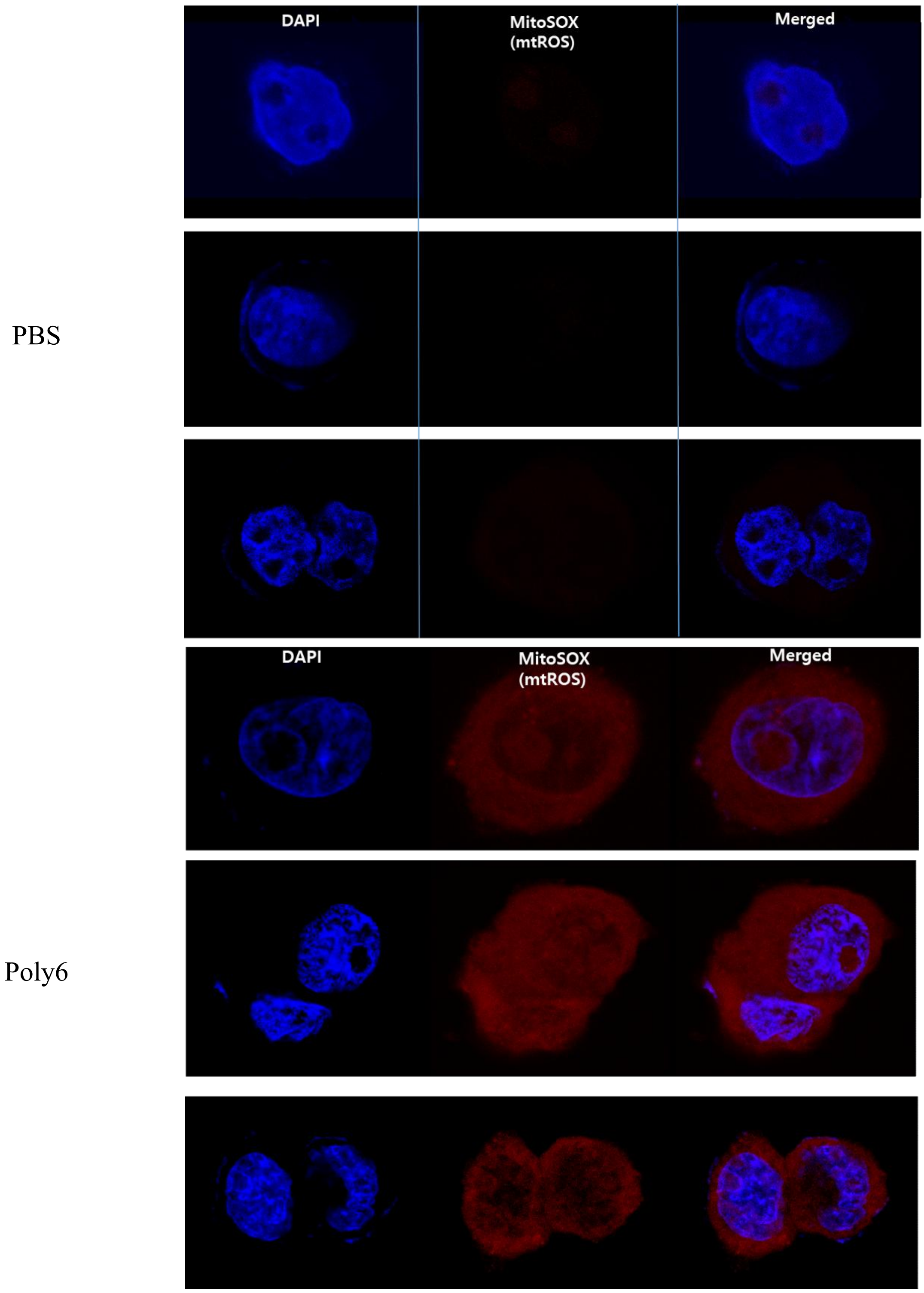

Supplementary Figure 8. Poly6–induced mitochondrial superoxide generation Confocal images of HepG2-2.15 cells showing elevated mitochondrial superoxide following administration of Poly6 for 12 h.

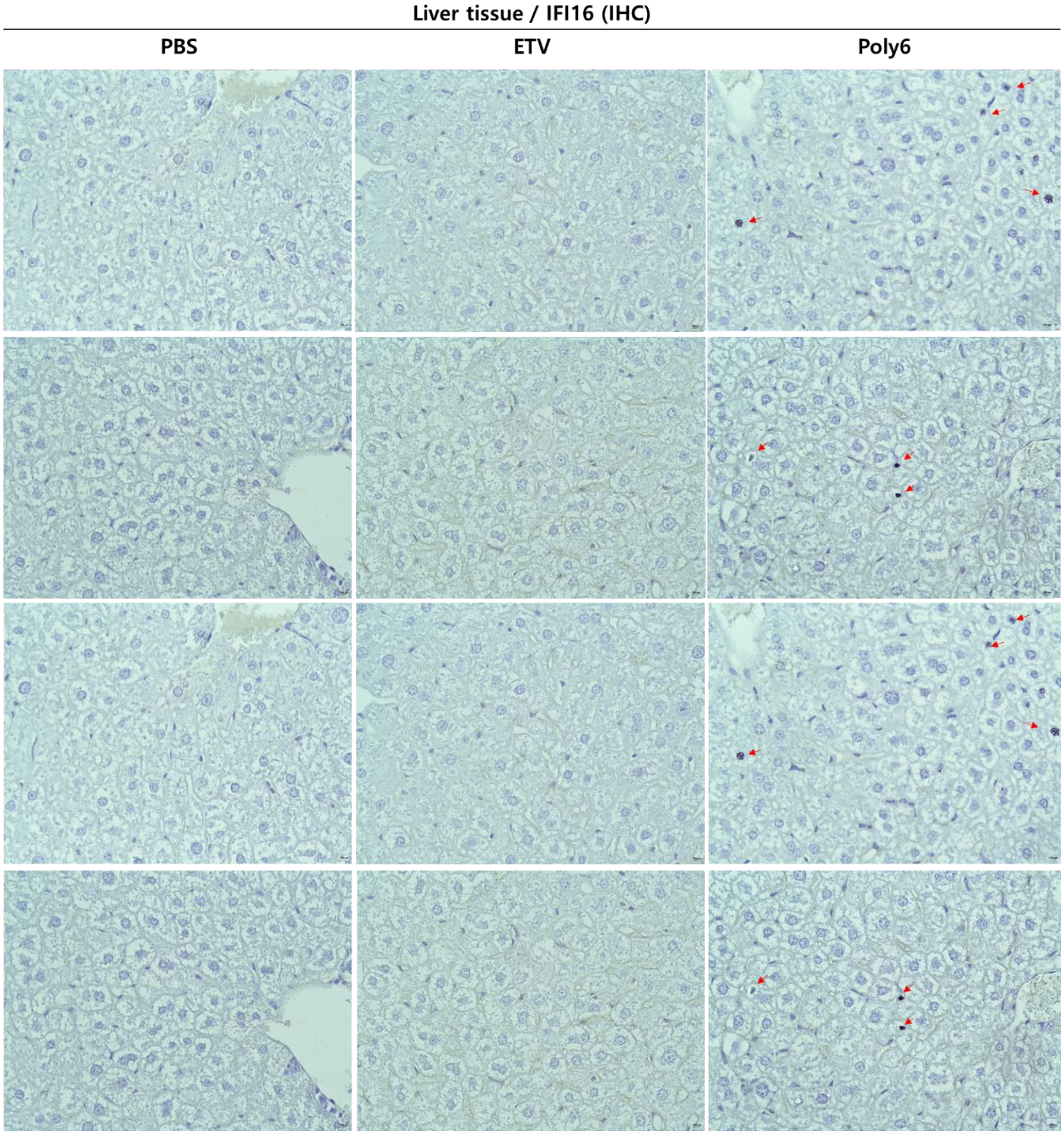

Supplementary Figure 9. Analysis of IFI16 expression in mouse liver tissues via Immunohistochemical staining.

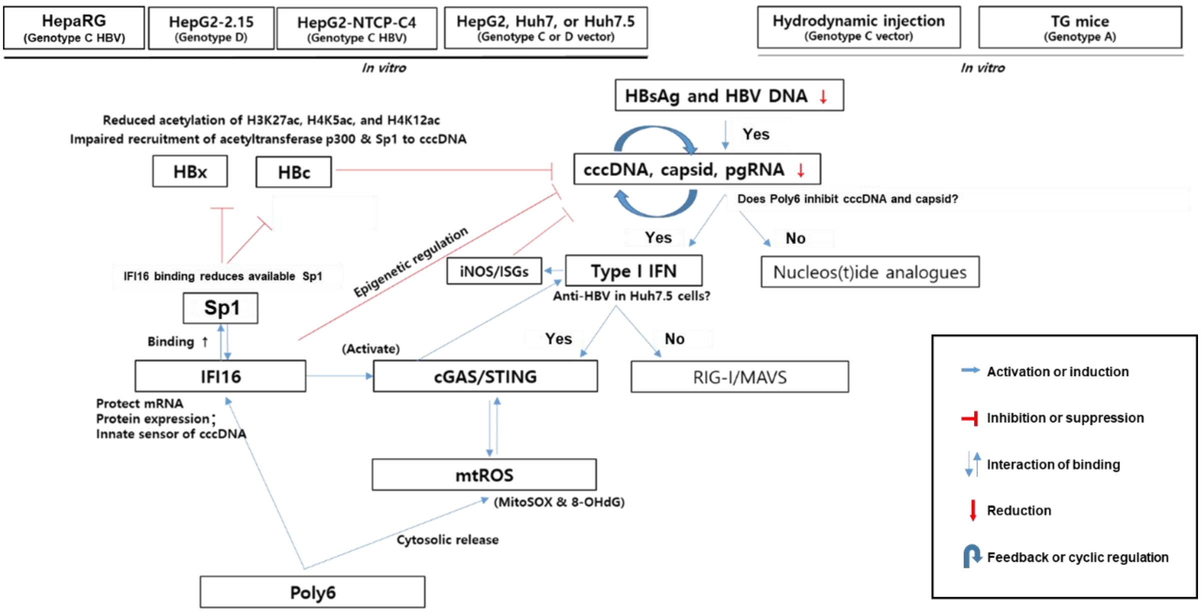

Supplementary Figure 10. A schematic model summarizing the proposed mechanism of Poly6-mediated inhibition of HBV replication.

Supplementary Table 1. List of Abbreviations

Abbreviation	Definition
cccDNA	Covalently closed circular DNA
cGAS/STING	Cyclic GMP-AMP synthase/stimulator of interferon genes
CHB	Chronic hepatitis B
DC	Dendritic cell
DS	Double-stranded
ETV	Entecavir
HBcAgs	HBV core antigens
HBsAgs	HBV surface antigens
HBx	HBV X protein
ICR mice	Institute of Cancer Research mice
IHC	Immunohistochemistry
LAM	Lamivudine
mtROS	mitochondrial ROS
NAs	Nucleot(s)ide analogs
NTCP	Sodium taurocholate cotransporting polypeptide
PBS	Phosphate-buffered saline
pgRNA	Pregenomic RNA
RC	Relaxed circular
ROS	reactive oxygen species
SS	Single-stranded
TG mouse	Transgenic mouse
8-OHdG	8-hydroxy-2'-deoxyguanosine

Supplementary Table 2.

Raw Ct Table

Figure 1F (left panel, PSAD treated)

Sample	Replicate #	Ct (cccDNA)	Ct (GAPDH)	ΔCt	ΔΔCt	Fold Change
Ctrl (PBS)	1	16.48	15.45	1.024431121179	-0.178189421559	1.13146301081506
	2	16.99	15.87	1.119956975894	-0.082663566844	1.05897135620998
	3	16.83	15.37	1.463473531142	0.260852988404	0.83459432254608
ETV	1	17.26	16.40	0.868232436106	-0.334388106632	1.26084253153999
	2	17.38	16.57	0.815220459827	-0.387400082912	1.30803403745929
	3	17.64	16.83	0.810238473003	-0.392382069735	1.31255881425293
poly10	1	16.77	15.26	1.514846713829	0.312226171090	0.80539801902176
	2	16.91	15.27	1.640480883466	0.437860340728	0.73822866318805
	3	16.96	15.24	1.722962783860	0.520342241121	0.69720641983100
poly20	1	17.02	15.43	1.581518798483	0.378898255744	0.76902464822019
	2	17.43	15.33	2.106662417884	0.904041875145	0.53438748444229
	3	17.89	15.89	1.997957614235	0.795337071497	0.57620853221884

Figure 1F (right panel, T5 exonuclease treated)

Sample	Replicate #	Ct (cccDNA)	Ct (GAPDH)	ΔCt	ΔΔCt	Fold Change
PBS-24 h	1	18.92	15.98	2.9348234138367	0.0091494739861	0.99367813550938
	2	18.89	15.97	2.9165244658644	-0.0091494739861	1.00636208472815
ETV-24 h	1	18.97	15.85	3.1279184299929	0.2022444901423	0.86919724679068
	2	18.88	15.83	3.0490635373280	0.1233895974775	0.91802821677907
Poly6-24 h	1	18.73	15.78	2.9494488489465	0.0237749090960	0.98365553309790
	2	18.74	15.89	2.8473024562164	-0.0783714836341	1.05582555015173
PBS-48 h	1	18.98	15.84	3.1446004027210	0.0426267106481	0.97088564855716
	2	18.97	15.91	3.0593469814247	-0.0426267106481	1.02998741559947
ETV-48 h	1	18.82	15.75	3.0739152656909	-0.0280584263819	1.01963897558481
	2	18.69	15.79	2.8986136511018	-0.2033600409711	1.15137679466271
Poly6-48 h	1	19.28	15.20	4.0798486428685	0.9778749507957	0.50772705704122
	2	19.28	15.13	4.1438944465906	1.0419207545178	0.48568042487261

Figure 1H (PSAD treated)

Sample	Replicate #	Ct (cccDNA)	Ct (18s)	mean Ct (18s)	ΔCt	ΔΔCt	Fold Change
ctrl	1	27.45	24.63	24.61	2.838558737706	-0.123669389577	1.0895024113979
	2	27.67	24.60	24.61	3.058007914626	0.095779787343	0.9357663220987
	3	27.36	24.47	24.42	2.940488891741	-0.021739235542	1.0151825919116
	4	27.43	24.37	24.42	3.011856965061	0.049628837777	0.9661848673804
ETV	1	27.41	24.47	24.43	2.984908110896	0.022679983612	0.9844023564295
	2	27.31	24.39	24.43	2.878508981769	-0.083719145515	1.0597464587685
	3	27.58	24.66	24.61	2.970334032605	0.008105905321	0.9943971693114
	4	27.49	24.56	24.61	2.882234533560	-0.079993593723	1.0570133468909
POLY6	1	28.16	24.12	24.51	3.653933687485	0.691705560201	0.6191214891024
	2	28.14	24.90	24.51	3.634088642669	0.671860515386	0.6276966805363
	3	27.43	24.47	24.38	3.047109919096	0.084881791812	0.9428617867676
	4	27.41	24.29	24.38	3.125259095200	0.163030967916	0.8931466804644

Supplementary Materials
- Western blot raw data

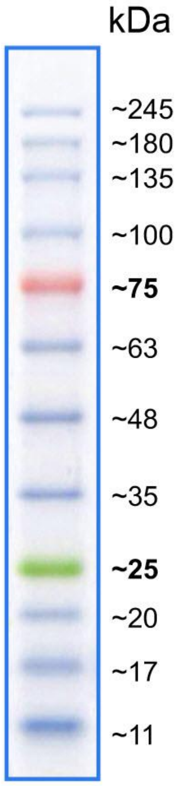

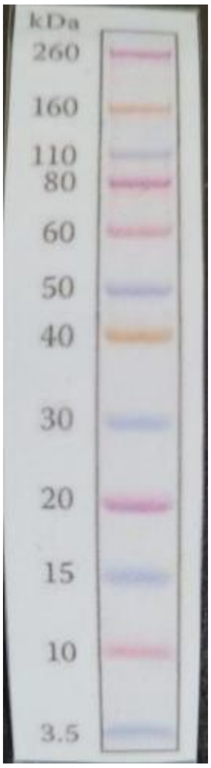

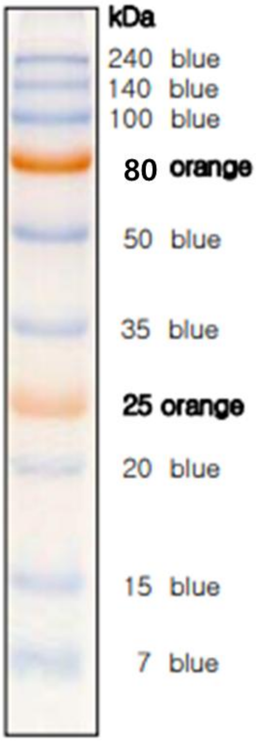

* Three different protein markers were used in this study

* Two different gel imagers (GE Amersham Imager AI680 & LAS2000) were used in this study.

Fig. 1D

**HBsAg (LHB)**	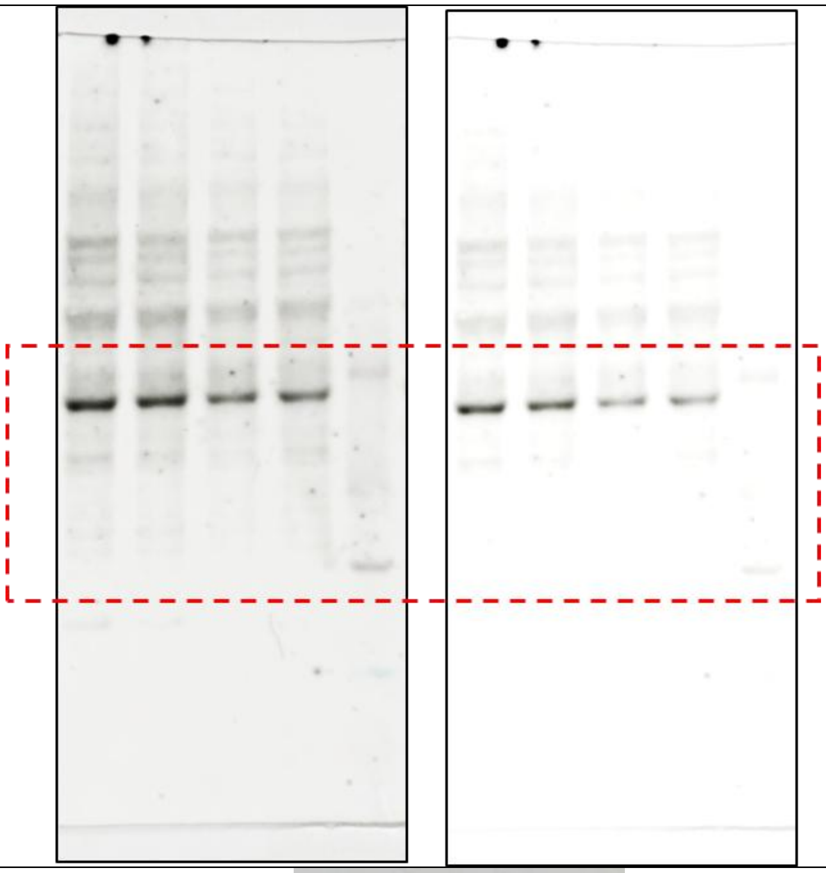
**GAPDH**	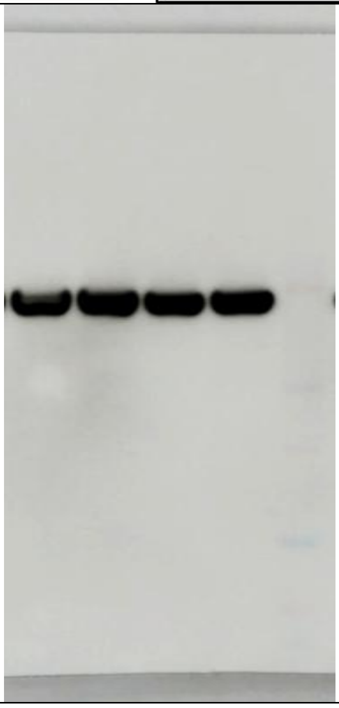

**HBc**	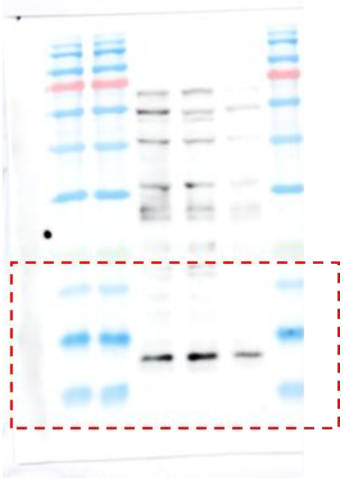
**GAPDH**	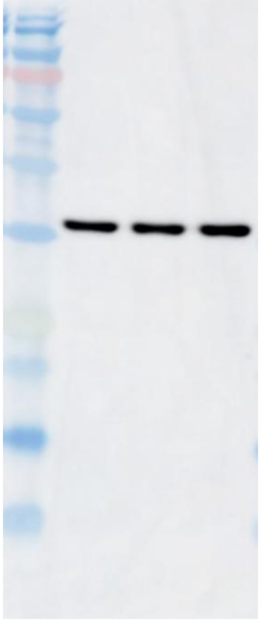

Fig. 1I

**Capsid**	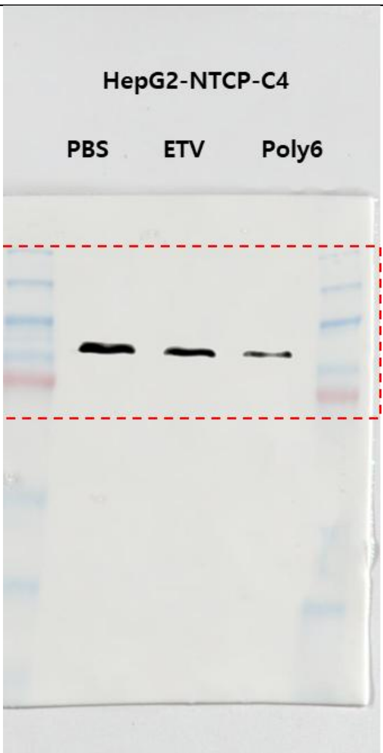
**GAPDH**	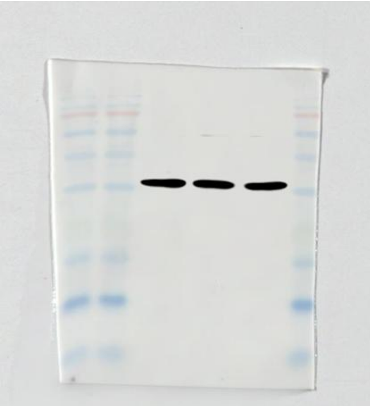 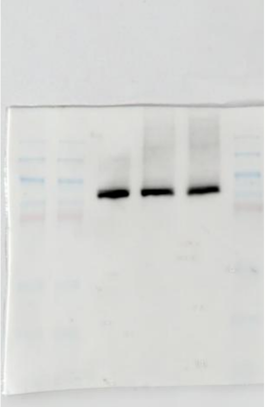

Fig. 3F

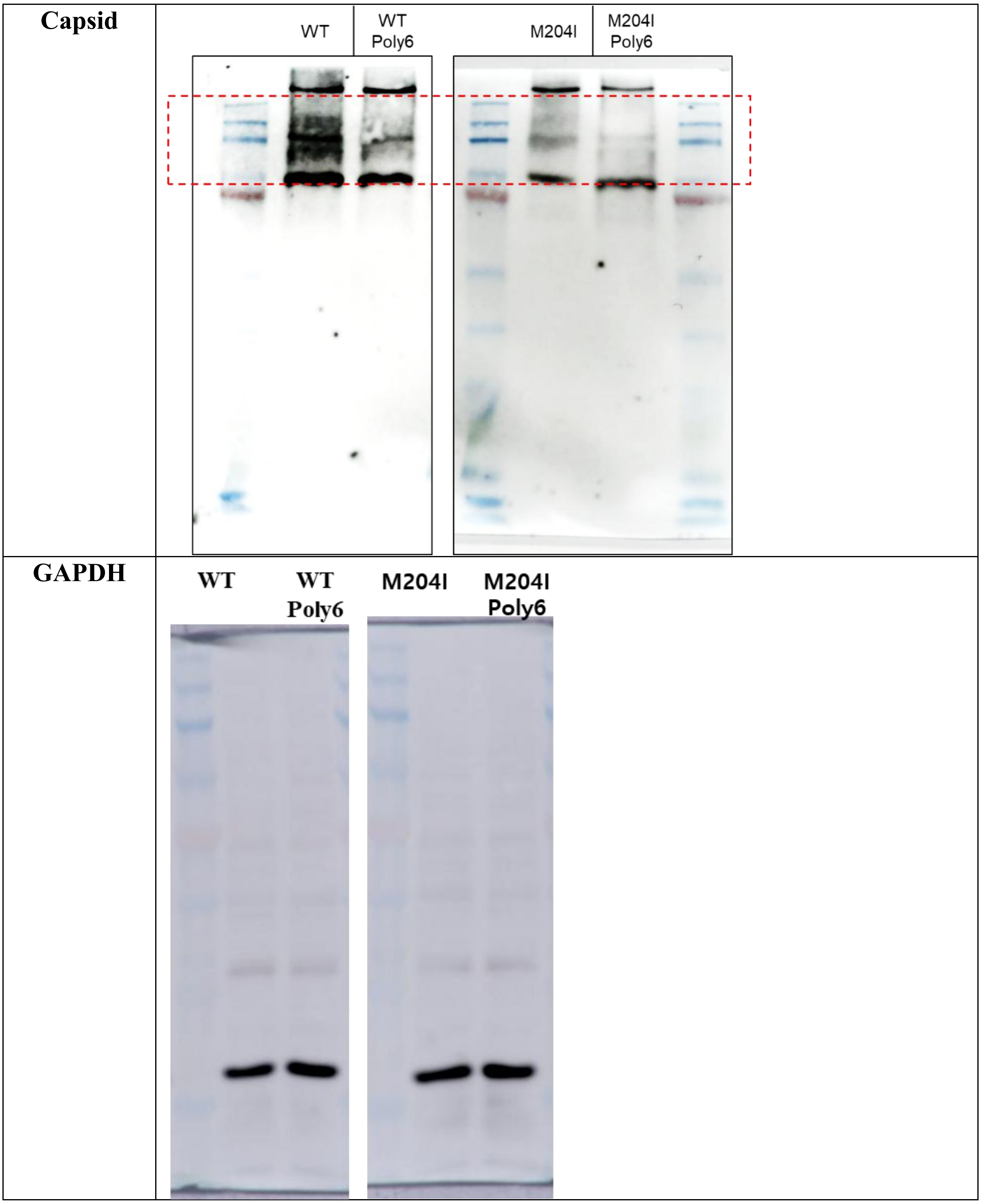
Capsid
WT
WT Poly6
M204I
M204I Poly6
GAPDH
WT
WT Poly6
M204I
M204I Poly6

Fig. 4E

**phospo-IRF3**	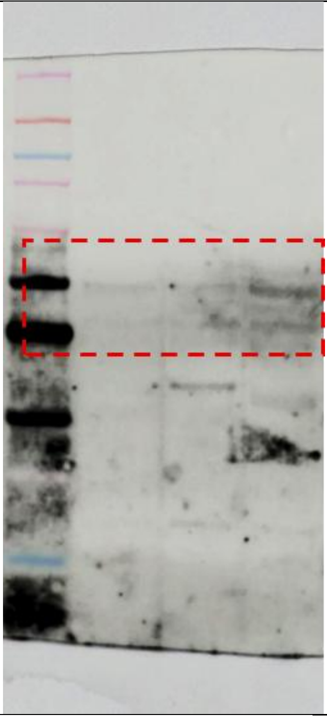
**IRF3**	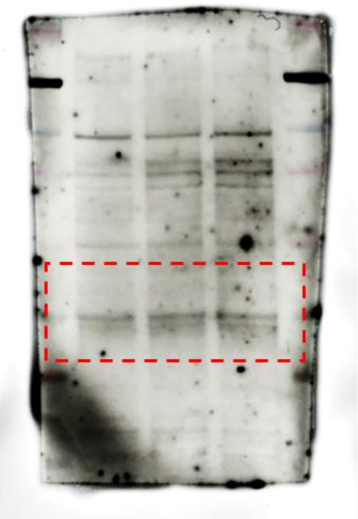
**GAPDH**	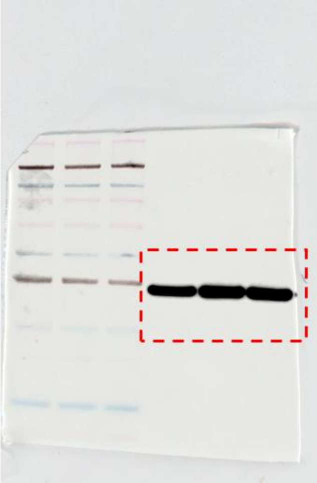

Fig. 5F

**Phospho-IRF3**	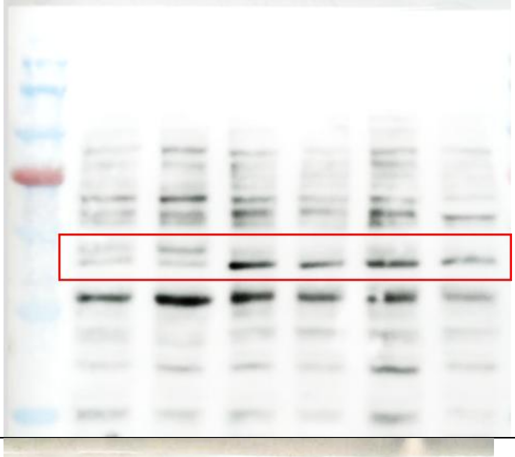
**IRF3**	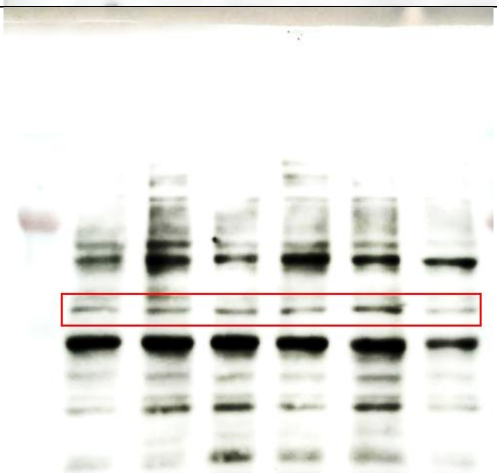
**STAT1**	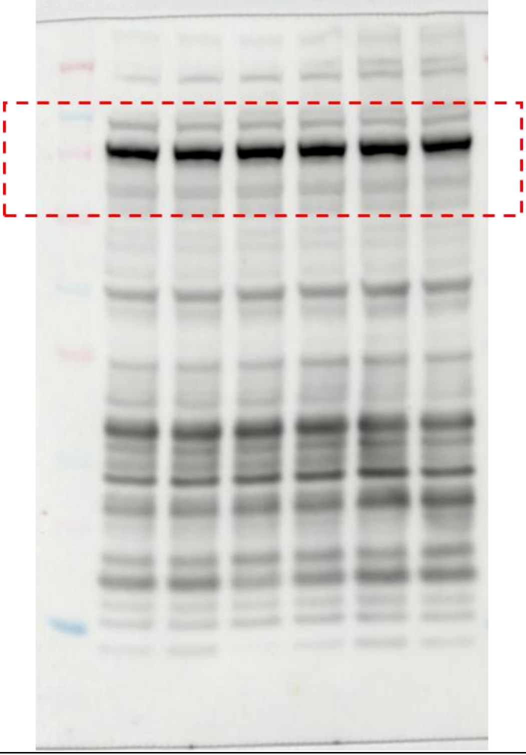

**Phospho-STAT1**	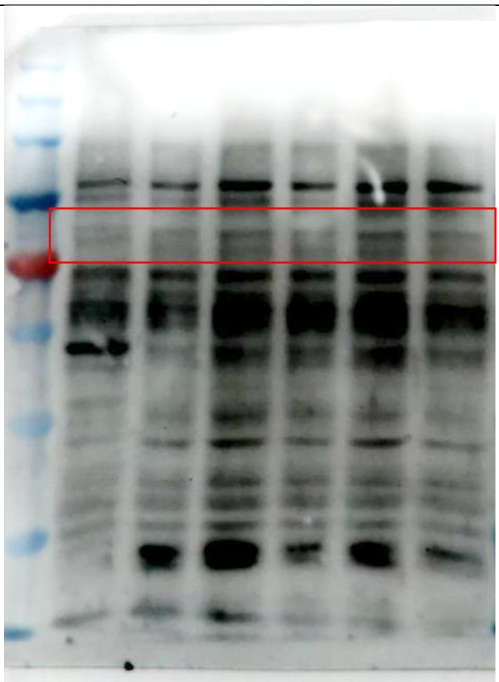
**GAPDH**	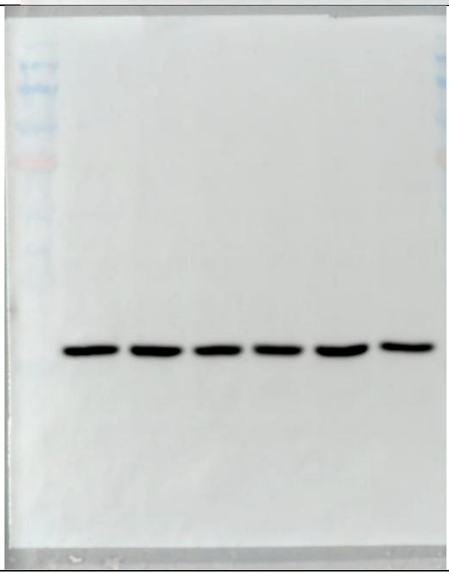

	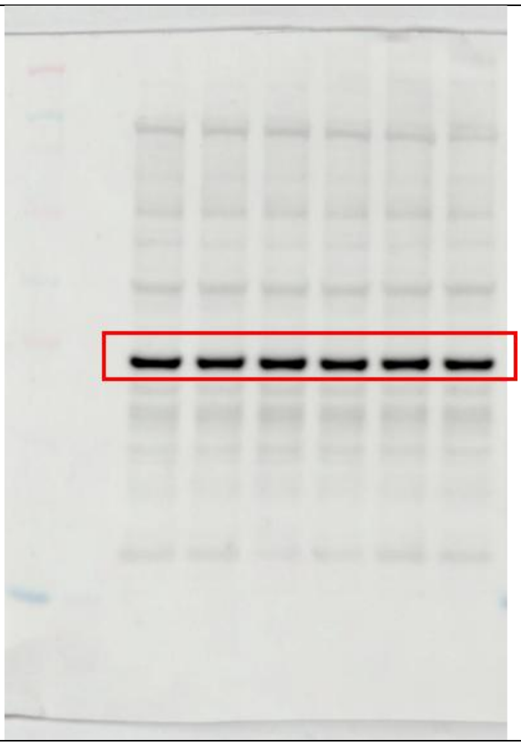	

Fig. 6A

IFI16

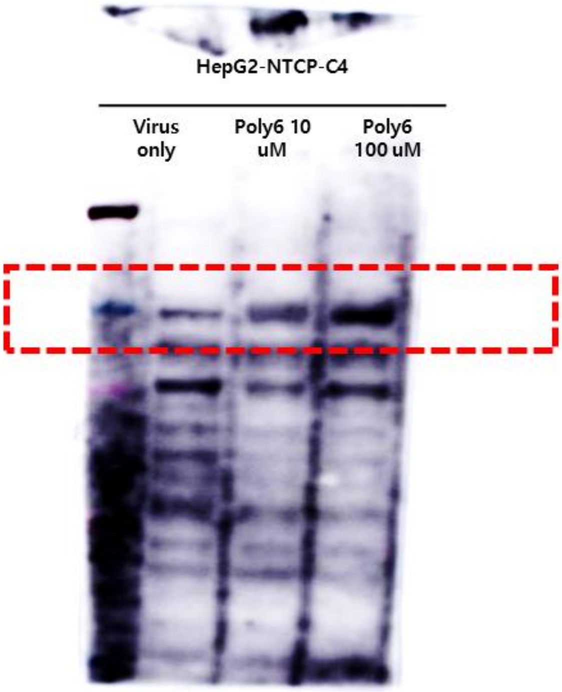

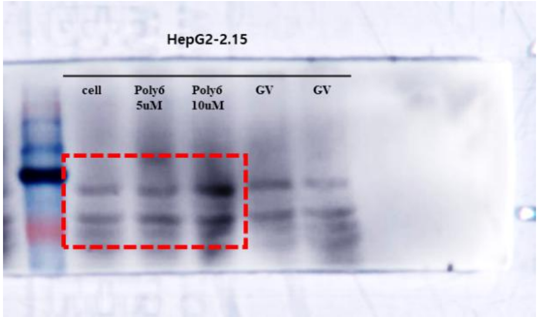

GAPDH

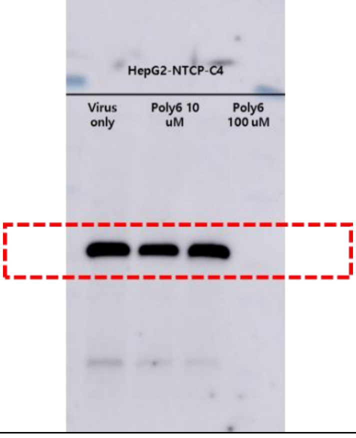

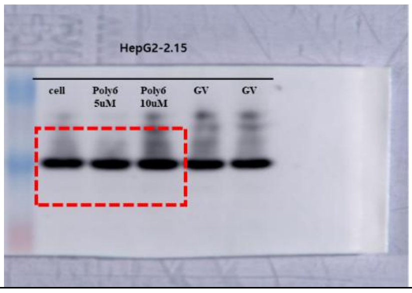

Fig. 6D

**Phospho--STAT1**	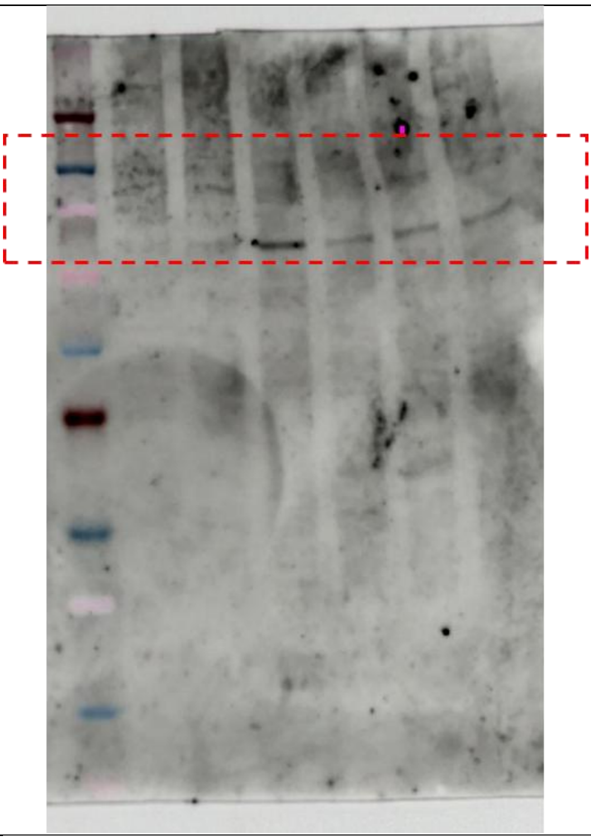
**STAT1**	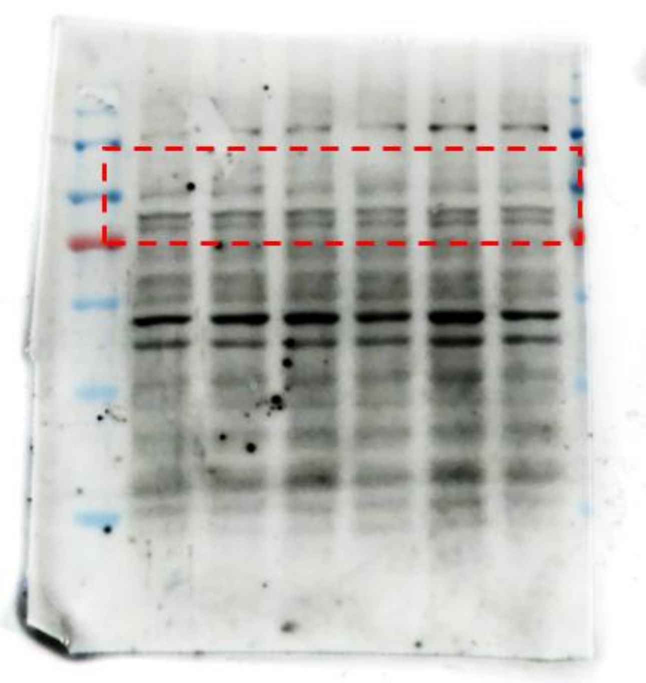

**Phospho--IRF3**	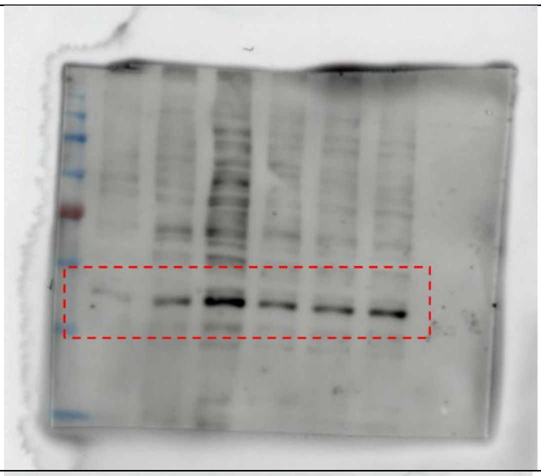
**IRF3**	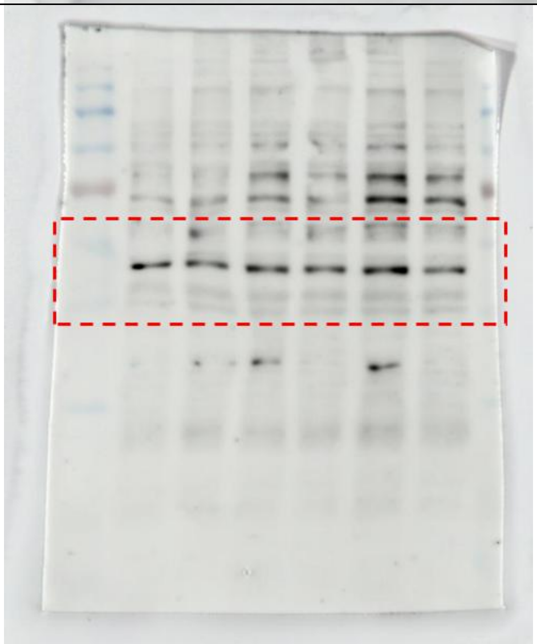
**GAPDH**	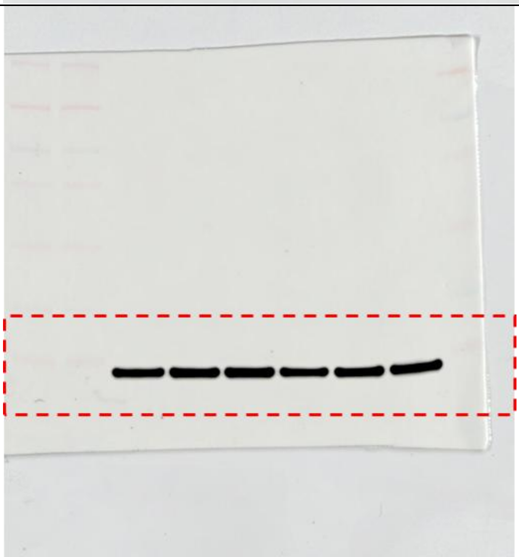

Fig. 6F

**IP: IFI16**<br>**IB: Sp1**	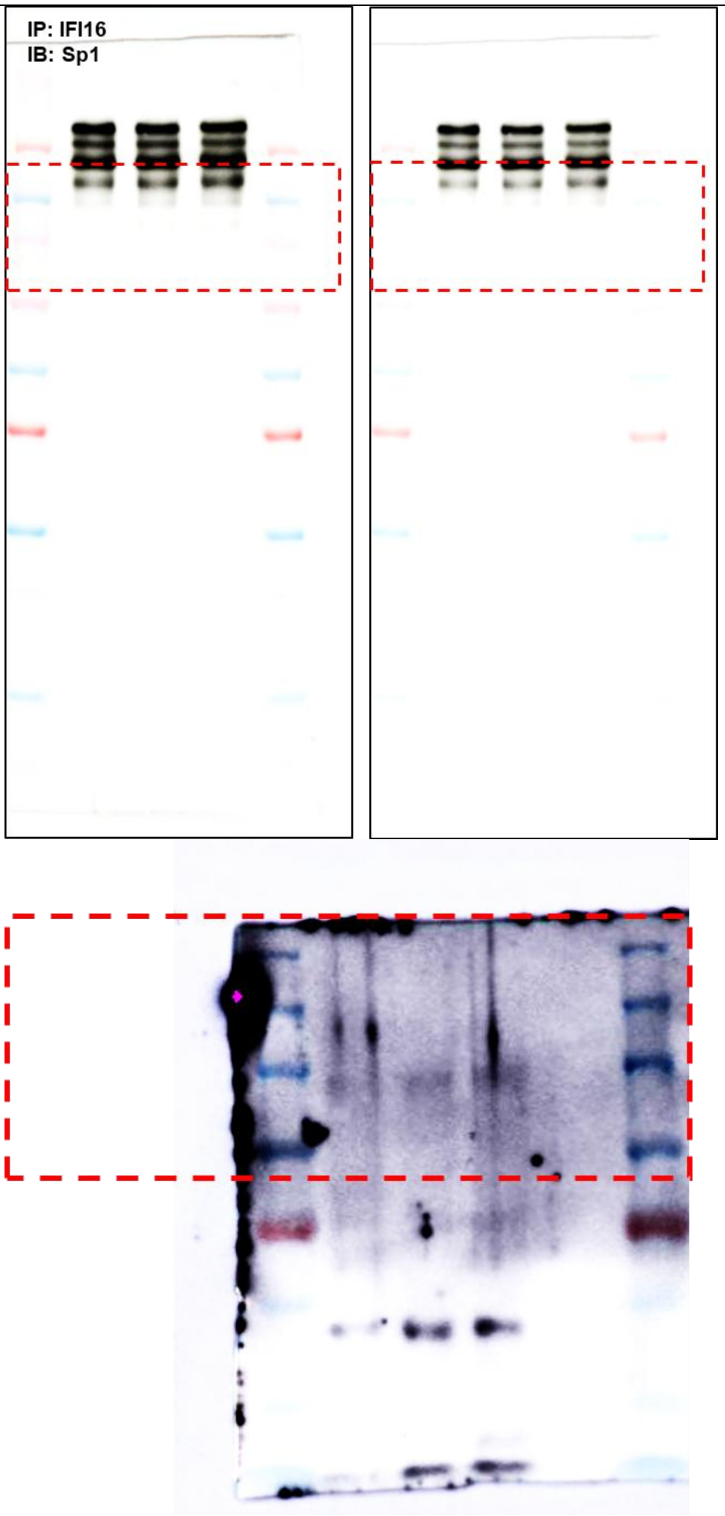

**GAPDH (input)**	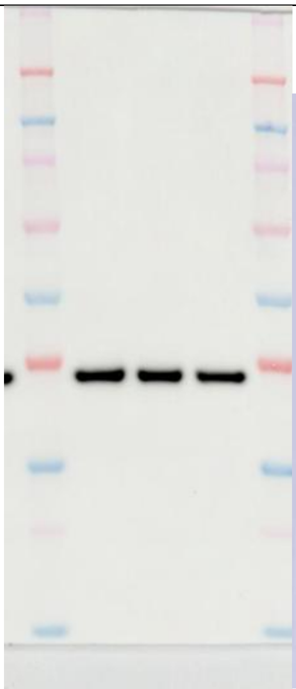 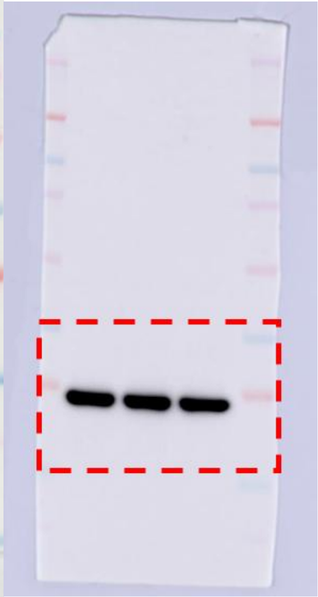
**Sp1 (input)**	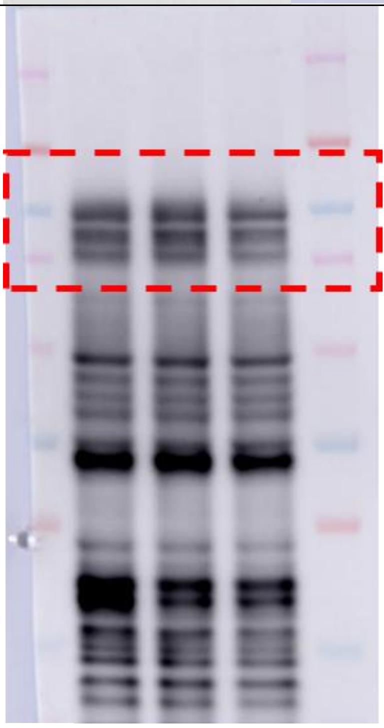 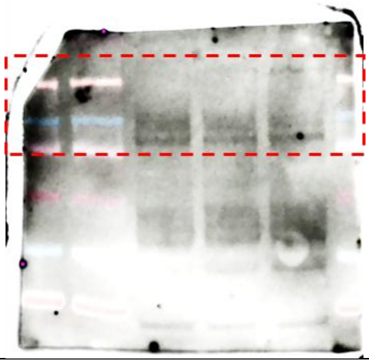

Fig. S3

**HBcAg**	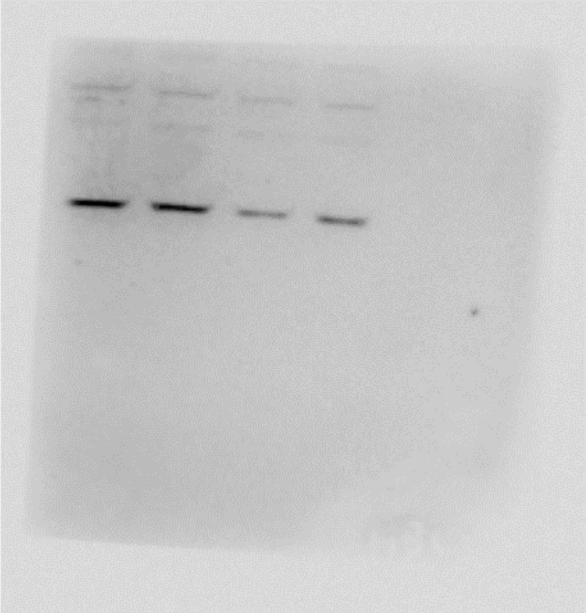
**GAPDH**	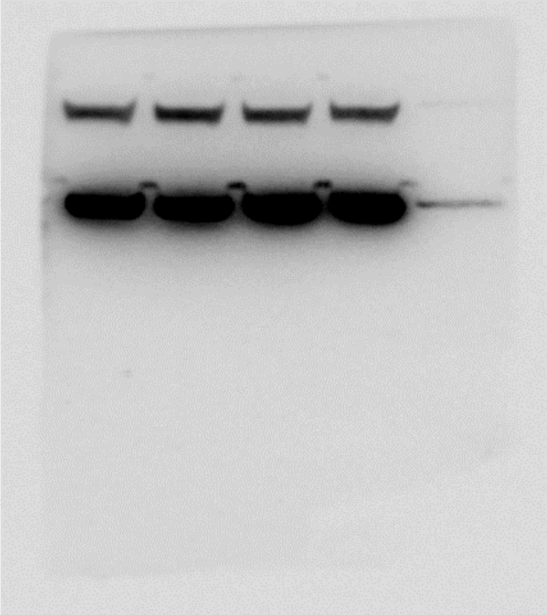

1. Choi Y-M, Kim H, Lee S-A, Lee S-Y, Kim B-J. 2020. A telomerase-derived peptide exerts an anti-hepatitis B virus effect via mitochondrial DNA stress-dependent type I interferon production. Frontiers in immunology 11:652.
